# Amplified intracellular Ca^2+^ for synergistic anti-tumor therapy of microwave ablation and chemotherapy

**DOI:** 10.1186/s12951-019-0549-0

**Published:** 2019-12-02

**Authors:** Jian-ping Dou, Qiong Wu, Chang-hui Fu, Dong-yun Zhang, Jie Yu, Xian-wei Meng, Ping Liang

**Affiliations:** 1 Department of Interventional Ultrasound, Chinese PLA General Hospital, 28 Fuxing Road, Beijing, 100853 China; 2 Laboratory of Controllable Preparation and Application of Nanomaterials, CAS Key Laboratory of Cryogenics, Technical Institute of Physics and Chemistry, Chinese Academy of Sciences, Beijing, 100190 People’s Republic of China

**Keywords:** Intracellular Ca^2+^, Synergistic therapy, Ablation, Chemotherapy

## Abstract

**Background:**

Developing new strategies to reduce the output power of microwave (MW) ablation while keeping anti-tumor effect are highly desirable for the simultaneous achievement of effective tumor killing and avoidance of complications. We find that mild MW irradiation can significantly increase intracellular Ca^2+^ concentration in the presence of doxorubicin hydrochloride (DOX) and thus induce massive tumor cell apoptosis. Herein, we designed a synergistic nanoplatform that not only amplifies the intracellular Ca^2+^ concentration and induce cell death under mild MW irradiation but also avoids the side effect of thermal ablation and chemotherapy.

**Results:**

The as-made NaCl–DOX@PLGA nanoplatform selectively elevates the temperature of tumor tissue distributed with nanoparticles under low-output MW, which further prompts the release of DOX from the PLGA nanoparticles and tumor cellular uptake of DOX. More importantly, its synergistic effect not only combines thermal ablation and chemotherapy, but also obviously increases the intracellular Ca^2+^ concentration. Changes of Ca^2+^ broke the homeostasis of tumor cells, decreased the mitochondrial inner membrane potential and finally induced the cascade of apoptosis under nonlethal temperature. As such, the NaCl–DOX@PLGA efficiently suppressed the tumor cell progression in vivo and in vitro under mild MW irradiation for the triple synergic effect.

**Conclusions:**

This work provides a biocompatible and biodegradable nanoplatform with triple functions to realize the effective tumor killing in unlethal temperature. Those findings provide reliable solution to solve the bottleneck problem bothering clinics about the balance of thermal efficiency and normal tissue protection.

## Background

Tumor therapies are developing quickly due to the development of modern technique and the unsatisfactory results of traditional therapies such as surgery and chemotherapy. Microwave (MW) ablation is a new and promising kind of thermal therapy with advantages of high efficiency, minimal invasion, and favorable efficacy [1]. However, its long-term survival outcomes are still not fully satisfactory, mainly because of the high rates of tumor recurrence and progression [2–4]. Non-lethal and insufficient thermal delivery is the most common cause for tumor progression after MW ablation, especially in the peripheral tumor area with lower temperature [5, 6]. Recently, different kinds of MW-sensitive agents and combined therapies of MW ablation and chemotherapy have been developed to enhance the therapeutic outcomes of MW ablation [7–10]. Nevertheless, all those efforts failed to solve the high recurrence in the peri-ablation areas of tumors. Solutions to induce tumor death in traditionally non-lethal temperature are highly desirable to achieve safe and effective antitumor therapy.

Typically, the best known biological response to MW therapy is thermal effect resulting from frictional heating of ions and polar molecules within cells in response to the oscillating electromagnetic field of MW [11]. Nevertheless, its biologic influence on ions movements deserves more attention with lots of potential benefits.MW has electromagnetic wave with frequencies of ≥ 300 MHz. Dipole molecule rotation and ionic polarization in the electromagnetic field have been shown to enhance transmembrane movement and interfere homeostasis of ions inside or outside of cells [9, 12–14]. Of all the intracellular or extracellular ions, Ca^2+^ is crucial and attractive as a pleiotropic second messenger involved in controlling many important physiological functions including cell proliferation, migration, and survival [15–17]. Moreover, its antitumor effect has gained lots of attention as a promising approach to trigger apoptosis in tumor cells [15, 18]. When an imbalance of intracellular Ca^2+^ occurs, mitochondria will take in Ca^2+^ from the cytoplasm to keep the intracellular calcium homeostasis. Then the overloading of Ca^2+^ in mitochondria would be followed by the damage of outer mitochondrial membrane and decrease of mitochondrial membrane potential(MMP) [19, 20]. Mitochondria is a primary energy factory for cells to keep working and is critical among many signal pathways involved in cell apoptosis, lipids and amino acids metabolism and so on.. Therefore, cell death is inevitable once mitochondrial dysfunction constantly occurs [21]. Studies focused on increasing the intracellular Ca^2+^ to trigger cell apoptosis have achieved promising results in many cancer treatments [22–24]. To the best of our knowledge, no systematic investigations have been conducted regarding regulation of intracellular Ca^2+^ concentration to enhance MW therapy under small thermal delivery.

Herein, a synergistic nanoplatform aimed at amplifying changes in intracellular Ca^2+^ was designed to investigate the synergic treatment of tumor cells by sensitizing them to MW irradiation under unlethal temperature conditions. Poly(lactic-co-glycolic acid) (PLGA) was selected as the carrier because it is one of the most common biodegradable polymeric compounds approved by the US FDA for use in drug delivery systems [25, 26]. Sodium chloride (NaCl), an ionic liquid with good MW sensitivity, was loaded into hollow PLGA nanoparticles to enhance MW efficiency by confining the collision of ions. Furthermore, DOX was loaded into the nanoparticles as we found that a low dose of DOX could significantly improve the intracellular Ca^2+^ concentration and induce massive tumor cell apoptosis under mild MW irradiation in low temperature. In this synergistic nanoplatform, DOX acted mainly as an accelerator of intracellular Ca^2+^ more than chemotherapy drugs. Additionally, a low dose of encapsulated DOX could reduce the toxic side effects.

**Scheme 1:**
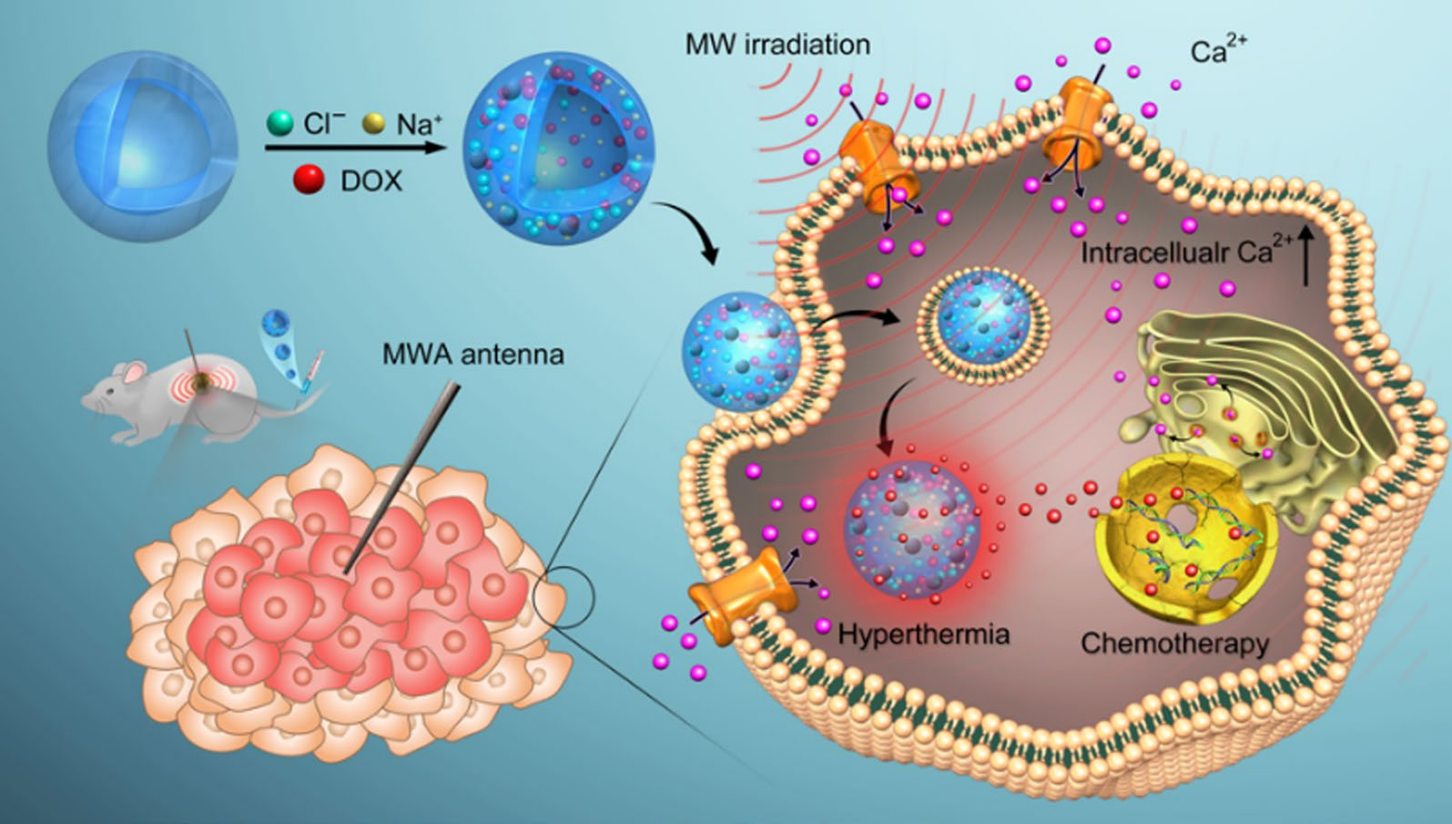
Schematic illustration of the multifunctional NaCl–DOX@PLGA nanoplatforms under mild MW irradiation. The thermal effect of the as-made nanoparticles could rise the temperature of the tumor and enhance DOX release. With the pretreatment of DOX and MW irradiation, intracellular Ca^2+^ concentration substantially increased, inducing more apoptosis. Hence, the multifunctional nanoplatform could realize effective tumor killing in a safe and controlled manner

On one hand, the as-made NaCl–DOX@PLGA nanoplatform selectively increased the conversion rate of MW energy into heat in tumor cells. On the other hand, it realized the high-efficient combination of thermal ablation and chemotherapy [27, 28]. More importantly, the presence of mild MW irradiation and DOX induced significant acceleration of intracellular Ca^2+^, which would break the homeostasis of tumor cells, decrease the MMP, finally induce the cascade of apoptosis under nonlethal temperature (Scheme 1). Thus, the biodegradable nanoplatform realized the triple effect of MW irradiation on tumor cells in a controlled, effective, and safe manner. This work provides highly compatible and feasible solutions for MW ablation to induce tumor death under non-lethal temperature and it has high potential for translation to the clinical setting.

## Materials and methods

### Materials

The mPEG–PLGA (mPEG, MW 1000, PLGA, lactide: glycolide 60:40, MW 100,000) were provided by Daigang Biomaterial Co., Ltd (Jinan, China). DOX·HCl was purchased from Huafeng United Technology Co., Ltd. Sodium chloride, Dimethyl sulfoxide (DMSO, 99%), and 1,4-dioxane were obtained from the Beijing Chemical Reagents Company. Polyvinylpyrrolidone (PVP, 95%) was purchased from Shantou Xilong Chemical Co., Ltd. Ethanol and aqueous ammonia were commercially available products. All reagents used in this work were of analytical grade without any further purification.

### Preparation of NaCl–DOX@PLGA drug-loaded nanoplatforms

In 4 mL DCM, 200 mg mPEG-PLGA was dissolved. A 0.5 mL aliquot of an aqueous solution (inner water phase) containing 10 mg DOX·HCl and 3% NaCl was emulsified for 90 s in an ice bath. A probe type sonicator (180 W, 90 s) was used to form a homogeneous primary emulsion. 10 mL water containing 5% PVA was added to the emulsion, and the solution was emulsified for another 90 s in an ice bath by the sonicator (400 W, 90 s). Then the acquired emulsion is dropped into 40 mL water (outer water phase) containing 0.1% PVA agitated by a magnetic stirrer for 4 h to remove the DCM. Pure mPEG-PLGA nanoparticles (without DOX·HCl) were prepared under constant conditions with only the inner water phase components changing. Microcapsules were collected and washed by centrifugation at 12,000 rpm for 40 min.

### Characterization

The morphology and size of the NaCl@PLGA and NaCl–DOX@PLGA nanoparticles were measured using a model 4300 scanning electron microscope (Hitachi). A Zetasizer (Malvern Instruments Zetasizer Nano ZS90, UK) was used to measure the hydrodynamic zeta potential and size of the NaCl@PLGA and NaCl–DOX@PLGA nanoparticles at a temperature of 25 °C. The absorption spectra of DOX were acquired via a UV–vis spectrophotometer (Jasco V-570 UV/vis/NIR spectrophotometer, Shanghai, China). The Na, Cl, C, and O elements in the NaCl–DOX@PLGA nanoplatform were characterized using scanning electron microscopy (SEM) X-ray (energy-dispersive spectroscopy [EDS]). The progress of MW heating in vivo and in vitro was recorded by forward-looking infrared (FLIR) and thermal needle. An Olympus X71 optical microscope (Japan) was used to observe the fluorescence within stained paraffin sections. The in vitro MW heating effect of the NaCl–DOX@PLGA nanoplatforms was evaluated by measuring the temperature at different nanoparticle concentrations using less than 10 W for 1 min. Briefly, 2 mL NaCl–DOX@PLGA nanoparticles at high and low concentrations (11, 22 mg mL^−1^) were placed into a 12-well plate and irradiated with 10 W and 2450 MHz MW for 1 min. PLGA nanoparticles, deionized water, and saline solution (0.9%) were used as controls. The surrounding temperature away from the MW antenna was monitored using a thermal needle. The corresponding thermal progress imaging was recorded by FLIR.

The controlled-release of DOX from the NaCl–DOX@PLGA nanoplatform was investigated. NaCl–DOX@PLGA nanoparticles were dispersed in 0.1 M phosphate-buffered saline (PBS) (5 mg/mL) and placed onto a rotary platform at 37 °C for 48 h. The PBS solution used was pH 7, pH 5, and pH 7 in the MW-1, MW-2, and control groups, respectively. The nanoparticles in the MW-1 and MW-2 groups were irradiated by 2450 MHz MW and 5 W for 4 min, and the temperature increased to 55 °C. Subsequently, the solutions were continuously shaken by a shaking table to evenly mix the nanoparticles. A UV–vis spectrophotometer was used to examine the DOX concentration at 483 nm in the supernatant via centrifugation. The amount of DOX release over time was confirmed through a standard calibration curve.

### mechanism study for the NaCl–DOX@PLGA nanoparticles sensitizing tumors to MW irradiation

#### Influence on intracellular Ca^2+^ concentration

We first tested the influence of free DOX with MW irradiation, and then the role of NaCl–DOX@PLGA nanoparticles combined with MW irradiation, on intracellular Ca^2+^ concentration. Tumor cells were plated into 6-well plates (2 × 10^5^ per well) and cultured in a suitable environment for 24 h. The cells were divided into four groups: the control, free DOX, MW, and DOX + MW groups. Subsequently, 100 μL PBS and DOX (3 μmol/L) were incubated with the cells for 12 h. The cells were then washed twice with Hank's Balanced Salt Solution (HBSS, without Ca^2+^) to remove excess DOX. The washed cells were incubated with 5 μM Fluo-3/AM for 40 min at 37 °C. The cells in the MW and DOX + MW groups were irradiated with mild MW (4 W for 2 min) in the dark. The mean fluorescence intensity was analyzed using a flow cytometer (BD Bioscience) at 488 nm. DOX was then incorporated into the nanoparticles to reduce toxicity and increase the cellular accumulation. The experimental methods were similar with the abovementioned methods to illustrate the influence of NaCl–DOX@PLGA nanoparticles combined with MW irradiation on intracellular Ca^2+^ concentration. The only difference was the replacement of DOX with NaCl–DOX@PLGA nanoparticles (186 μg/mL).

#### Mitochondrial inner membrane potential

The lipophilic cation-staining agent 5,50,6,60-tetrachloro-1,10,3,30-tetraethylbenzimidazolylcarbocyanine iodide (JC-1) was selected to evaluate changes in the MMP. JC-1 emits a red fluorescence signal in the upper left quadrant of the fluorescence-activated cell-sorting (FACS) display (J-aggregate) when the membrane potential increases, and a green signal is emitted in the upper right and lower right quadrants of the FACS display when apoptosis occurs. Cells in a 12-well plate were cultured in a thermostat incubator at 37 °C in a humid atmosphere with 5% CO_2_. They were subsequently washed with PBS and divided into four groups: the control, positive, MW, and MW + NaCl–DOX@PLGA groups. The MW + NaCl–DOX@PLGA group had 1 mL NaCl–DOX@PLGA nanoparticles (186 μg/mL) added to the cell medium, and 1 mL DMEM was added to the other groups. All cells were cultured in a stable environment for 12 h. Subsequently, the cells in the MW and MW + NaCl–DOX@PLGA groups were treated with MW (4 W for 2 min) and were cultured for an additional 1 h. The cells in the positive group were incubated with CCCP (10 μmol/L) for 30 min. Subsequently, all cells were incubated at 37 °C for 20 min with 5 mg/mL JC-1. After two wash cycles with phosphate-buffered JC-1 solution, the green fluorescence of monomeric JC-1 and the red fluorescence of aggregate JC-1 were recorded as a function of time using a Fluostar Omega microplate reader. Excitation was fixed at 490 nm, and the emission was alternately collected at 530 and 590 nm. Data were expressed as ratio of red/green fluorescence after correction for DOX autofluorescence and/or energy transfer to JC-1.

The fluorescence intensity changes were also measured by flow cytometry to quantify changes in mitochondrial inner membrane potential caused by MW and the presence of nanoparticles. The experimental methods were similar with the JC-1staining methods as previously mentioned. The differences included cells were cultured in a 6-well, rather than a 12-well, plate at 37 °C, and the influence of MW, MW + DOX, and MW + NaCl–DOX@PLGA nanoparticles on mitochondrial inner membrane potential was evaluated. The fluorescence intensity of the cells was analyzed using a flow cytometer.

#### Cellular uptake and measurement of cellular viability

To study the cellular uptake of PLGA nanoparticles, HepG2 cells were seeded into 12-well plates and cultured in a suitable environment for 24 h. The medium was then replaced with 1 ml of fresh medium containing nanoparticles encapsulated with rhodamine 6G (30 μg/ml). All samples were rinsed three times with PBS after incubation with the cells for 8 h. Then the cells were further incubated for 10 min in the medium containing 4′,6-diamidino-2-phenylindole(DAPI). After being washed with PBS three times, the cells were observed using a fluorescence microscope (IX73IPF, Olympus Corporation, Japan).

HepG2 cells were seeded in 6-well plates. After 24 h incubation, the cells were divided into five groups, with three samples in each group. The cells in the MW + NaCl–DOX@PLGA and NaCl–DOX@PLGA groups were cultured with NaCl–DOX@PLGA nanoparticles (186 μmol/L) for 12 h. The cells in the MW + DOX group were cultured with free DOX (3 μmol/L) for 12 h. Twelve hours later, the cells in the MW, MW + DOX, and MW + NaCl–DOX@PLGA groups were irradiated with mild MW (4 W for 2 min) and were subsequently placed back in the thermostat incubator. The cells were harvested after 4 h and stained with the Annexin V-FITC/PI apoptosis detection kit, according to manufacturer's instructions. Apoptotic cells were detected and analyzed by flow cytometry.

### Effect of EGTA, 2-APB, TMB-8, and thapsigargin on MW and nanoparticle-increased intracellular Ca^2+^ concentration

An increase in intracellular Ca^2+^ concentration primarily originates from an increased release of intracellular Ca^2+^ from the endoplasmic reticulum (ER) Ca^2+^ stores and plasmalemmal Ca^2+^ entry from store-operated Ca^2+^ entry in hepatocytes. We explored the Ca^2+^ pathway in response to MW and nanoparticles in HepG2 cells. Extracellular Ca^2+^ chelator, EGTA (0.5 mM), store-operated Ca^2+^ influx inhibitor, 2-aminoethoxydiphenyl borate (2-APB, 50 μM), ER Ca^2+^ release inhibitor, 3,4,5-trimethoxybenzoic acid 8-(diethylamino) octyl ester (TMB-8, 100 nM), and sarco/ER-Ca^2+^-ATPase inhibitor(SERCA) inhibitor thapsigargin (0.1 mM) were added to the cells to remove Ca^2+^. After 12 h culture, the cells were stained with Fluo-3 for 40 min and subsequently treated with mild MW irradiation (4 W for 2 min). The fluorescence intensity was measured with a FACSCalibur flow cytometer (Becton Dickinson). The results of 10,000 fluorescent events by CellQuest were analyzed with FCS Express 4.0 (De Novo Software) and expressed as the mean fluorescence intensity of 10,000 cells.

#### In vitro cytotoxicity

The standard MTT assay was used in the HepG2 cells to estimate the biocompatibility of the NaCl–DOX@PLGA nanoparticles. The cell viability and morphology reflect the cytotoxicity of the NaCl–DOX@PLGA nanoparticles. The tumor cells were plated into 96-well plates (1 × 10^4^ per well) and cultured in a suitable environment for 24 h. Subsequently, 100 μL PBS and NaCl–DOX@PLGA nanoparticles were added to the plates. Nanoparticles of different concentrations (25, 50, 75, 150, 300, 400, and 600 μg/mL) were incubated with the cells for different durations. Subsequently, the cells were incubated for 24 h, and the cell viability was represented as the absorbance of formazan at 490 nm. The control, PBS treated, cells were considered as 100% viable.

#### In vitro hemolysis test

The hemolysis test was performed using rabbit's heart blood to evaluate the cytotoxicity of NaCl–DOX@PLGA nanoparticles in vitro. Five milliliters of blood was obtained from the rabbit heart, and 0.2 mL anticoagulant agent was added. The blood samples were washed with PBS to remove external and lysed red blood cells. After removal of the supernatant, 1 mL blood was diluted to 50 mL with PBS. Subsequently, 0.5 mL cells were mixed with NaCl–DOX@PLGA nanoparticles (0.5 mL) diluted in PBS at concentrations of 25, 50, 75, 150, 300, 400, and 600 μg mL^−1^. The positive and negative control groups composed of mixtures of 0.5 mL cells and 0.5 mL deionized water and 0.5 mL cells and 0.5 mL PBS, respectively. Three parallel experiments were performed in duplicate for each group. The mixtures were centrifuged under room temperature for a 3 h incubation period. The absorbance of the supernatant was measured at 570 nm via UV–vis.

#### Safety injection dose evaluation in vivo

Twenty healthy female nude mice were randomly and equally divided into four groups to investigate the safety injection dose of NaCl–DOX@PLGA nanoparticles. The mice were injected with NaCl–DOX@PLGA nanoparticles at different doses of 20 to 110 mg kg^−1^ (20, 60, and 110 mg kg^−1^, dispersed in PBS) via the tail vein and killed after 14 days. PBS-treated mice were used as the control group. The blood was collected for blood biochemistry and blood routine examination. Major organs, including the heart, spleen, liver, lung, and kidney, were harvested for histological examination.

### Antitumor efficacy evaluation of NaCl–DOX@PLGA in vivo under mild MW irradiation

We randomly divided 48 HepG2-tumor-bearing female nude mice into the following six groups: PBS, PBS + MW, DOX, NaCl–DOX@PLGA, NaCl–DOX@PLGA + MW, and NaCl@PLGA + MW groups. The injection dose was 110 mg kg^−1^ except for the DOX group, which was 10 mg kg^−1^, and the abovementioned materials were injected into the mice via the tail vein. The MW antenna was inserted into the center of the tumor under real-time ultrasound guidance (Additional file 1: Figure S1) in the MW groups (PBS + MW, NaCl–DOX@PLGA + MW, and NaCl@PLGA + MW) at 6 h post-injection. The power was 2450 MHz, and the output energy was set at 2 W for 1.5 min. The temperature changes during MW heating in vivo were recorded using FLIR imaging. During the investigation, body weight and tumor size were carefully observed for 2 or 3 days.

Three mice in every group were killed 3 days after ablation to obtain tumor tissues to analyze the therapeutic effect at an early stage. Histological analysis was performed to analyze coagulative necrosis. Cell apoptosis and proliferation in the peripheral ablation area was assessed by TUNEL and PCNA staining. The TUNEL assay and PCNA staining (Roche, Basel, Switzerland, and California, USA, respectively) were performed on the basis of manufacturer's instructions. The percentages of TUNEL-positive and PCNA-positive nuclei were calculated as follows: four randomly chosen fields per section corresponding to at least 50 cells were examined at high magnification (400 ×). The mice were killed and marked as state of “death” if the tumor size in any one direction reached 20 mm. The survival rates of mice were measured similarly. The tumors and major organs of the mice in each group were excised and stained with hematoxylin and eosin (H&E) for histopathological analysis.

## Results and discussion

### Synthesis and characterization of NaCl–DOX@PLGA nanoparticles

The successful synthesis of NaCl@PLGA was confirmed by measuring the hydrodynamic diameter and zeta potential of the nanoparticles (Additional file 1: Figure S2a, b). The confinement effect of the nanoparticles can be used to accelerate the movement of encapsulated ions or molecules under the MW field, producing more heat and significantly increasing the surrounding temperature. This effect has been used to enhance tumor ablation [7, 29]. NaCl, a known ideal MW-sensitive material, can be loaded into the PLGA nanoparticles by a simple microemulsion method. The MW heating effect of 22 mg mL^−1^ NaCl@PLGA nanoparticles was tested. Additional file 1: Figure S3 shows that the temperature in the NaCl@PLGA group showed a more rapid increase than that in the PLGA and NaCl solution groups. The largest temperature gap was approximately 8.3 °C between the NaCl@PLGA and NaCl groups. Those results indicated that the PLGA nanoparticles were suitable to load NaCl to enhance MW ablation.

**Fig. 1:**
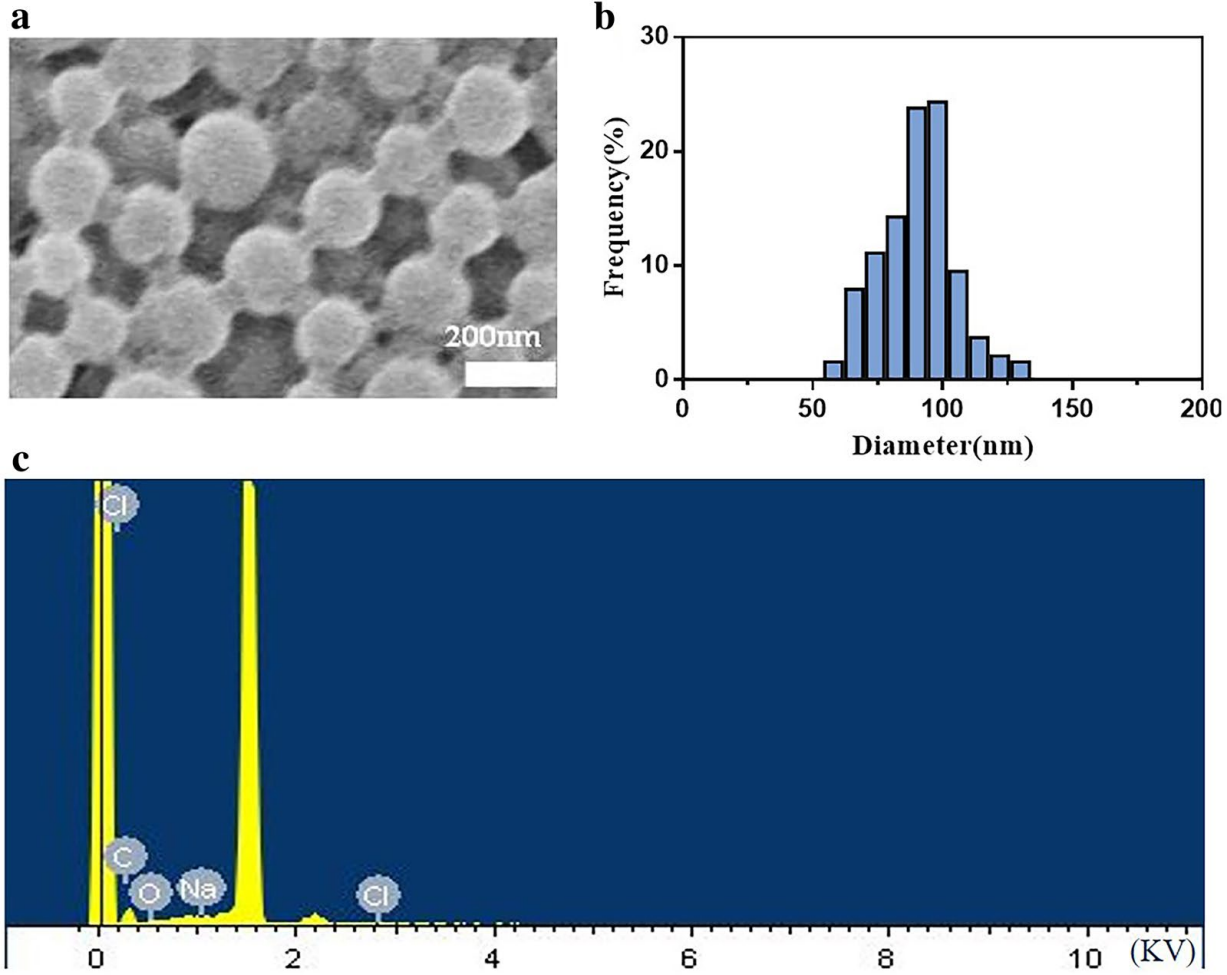
**a** The SEM image of NaCl–DOX@PLGA nanoparticles. **b** Distribution of particle size histograms of NaCl–DOX@PLGA nanoparticles. **c** EDS of the NaCl–DOX@PLGA nanoparticles

To realize the effect of sensitizing tumor cells to MW and improving the therapeutic efficiency of MW ablation, DOX was packaged into the PLGA nanoparticles. The NaCl–DOX@PLGA nanoparticles had a diameter of 100 nm, which was similar to the NaCl@PLGA nanoparticles. Figure 1a, b show SEM images of NaCl–DOX@PLGA nanoparticles; the SEM inset reveals the hollow structure of the PLGA nanoparticles. EDS was used to detect the elements contained in the nanoplatform. Figure 1c shows that the elements Cl, Na, C, and O were detected in the sample, which confirms the presence of DOX (C_27_H_29_NO_11_·HCl), NaCl, and PLGA (which is mainly composed of lactic acid [C_3_H_6_O_3_] and glycolic acid [C_2_H_4_O_3_]).

**Fig. 2:**
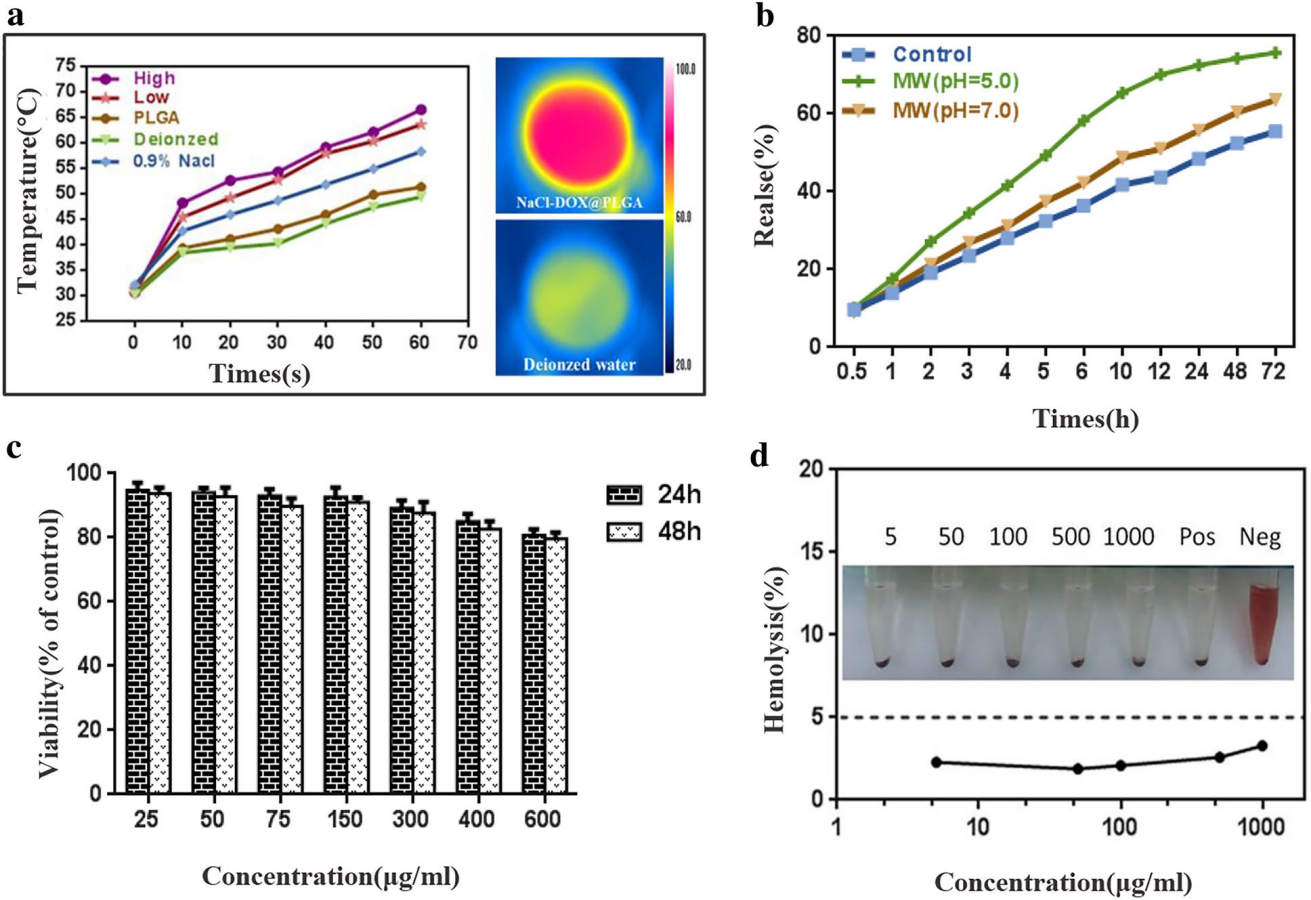
The MW heating properties, the release behavior of DOX at different pH conditions and the toxicity evaluation of the NaCl–DOX@PLGA nanoparticles. **a** The temperature changes of different groups. The right above FLIR image showed the temperature at the end of MW ablation in high group and the below image in the deionized group. **b** DOX release behavior of the NaCl–DOX@PLGA nanoparticles; **c** The viability of HepG-2 cells after being incubated with NaCl–DOX@PLGA nanoparticles (25,50,75,150,300,400 and 600 μg/mL, PBS as control). **d** Hemolysis ratio of NaCl–DOX@PLGA nanoparticles with different concentrations of 25,50,75,150,300,400 and 600 μg/mL, the inset represents the hemolysis conditions in tubes with different concentrations of NaCl–DOX@PLGA nanoparticles. *Pos* the positive control group, *Neg* the negative control group

As shown above, the NaCl@PLGA nanoparticles had an obvious hyperthermic effect due to the sensitivity of NaCl to MW in an enclosed space. To evaluate the heating effect of the NaCl–DOX@PLGA nanoparticles in vitro, the energy output of the 2450 MHz MW equipment was set at 10 W for 1 min to heat 2 mL of the nanoparticles at different concentrations. Figure 2a shows the heat effects of the nanoparticles. The MW heating effect increased with an increase in NaCl–DOX@PLGA concentration. After a 60 s MW irradiation, the temperature change was highest (66.5 °C) where there was a high concentration of nanoparticles, lower (63.6 °C) where there was a low concentration of nanoparticles, and lowest (49.4 °C) in the deionized water group, in the absence of nanoparticles. Corresponding temperature changes were recorded every 10 s using a thermal monitoring needle and an FLIR imaging instrument. The results showed that the NaCl–DOX@PLGA nanoplatform achieved a successful MW heating effect in the simulated body fluid.

The loading capacities of DOX and NaCl in the PLGA nanoparticles were 29.1% ± 2.03% (w/w) and 3.1%, respectively. The temperature of the nanoparticles obviously increased because the nanoparticles were irradiated by MW. PLGA-based drug delivery systems have several therapeutic applications due to its biodegradability, biocompatibility, and sustained-release properties [25, 30]. The following three groups were designed to examine the MW-stimulated release properties of DOX from the as-made nanoparticles. 1) The control group: 22 mg NaCl–DOX@PLGA nanoparticles was dispersed into 1 mL phosphate-buffered saline (PBS) (0.1 M, pH 7.2) solution, and the released amount of DOX under constant shaking for 1 h at 37 °C was tested. 2) MW (pH = 5.0) group: 22 mg NaCl–DOX@PLGA nanoparticles were dispersed into 1 mL PBS, the solution was placed into a water bath at 37 °C and shaken continuously, treated for 4 min by MW 30 min after shaking. The supernatant of the solution was collected after MW irradiation to measure the released amount of DOX. 3) The MW (pH = 7.2) group: 22 mg NaCl–DOX@PLGA nanoparticles were dispersed into 1 mL PBS (0.1 M, pH 7.2) solution, and the following methods and MW output energy were same with the MW (pH = 5.0) group. The nanoparticles showed good drug release properties. The release of DOX from the control group was initially slow and reached 55.48% at 72 h. After MW irradiation, the release of DOX showed a different increase rate, particularly in the acid condition. Figure 2b shows that the amount of DOX released in the MW (pH = 5.0) group reached 65.43% after 10 h. Subsequently, the release decreased, and the final amount released was approximately 75.78% after 72 h. The results showed that DOX could be effectively released from the NaCl–DOX@PLGA after MW irradiation after uptake by tumor cells.

### *Cellular uptake and *in vitro cytotoxicity

The cellular uptake of nanoparticles was evaluated via in vivo fluorescence images. With 8 h of incubation of HepG2 cells with PLGA nanoparticles, nanoparticls were successfully transported into cells (Additional file 1: Figure S4). An MTT assay was used to investigate the cytotoxicity of NaCl–DOX@PLGA on HepG2 cells. In our previous work, NaCl@PLGA nanoparticles showed good biocompatibility, which was suitable for further experiments in vivo. After being loaded with DOX, the NaCl–DOX@PLGA nanoparticles displayed similar cytotoxicity with NaCl@PLGA. Figure 2c shows that the NaCl–DOX@PLGA nanoparticles exhibit a dose-dependent cytotoxicity towards HepG2 cells. The relative cell viability was > 80% in groups exposed to a concentration below 600 μg mL^−1^.

A hemolysis test was performed to further evaluate the potential cytotoxicity of NaCl–DOX@PLGA nanoparticles. Figure 2d shows the hemolysis ratio caused by different NaCl–DOX@PLGA nanoparticle concentrations. None of the hemolysis ratios in any experimental group exceeded 5%, and the mean value was only 2.44%, indicating that the NaCl–DOX@PLGA nanoparticles showed low toxicity. The inset in Fig. 2d shows the visual hemolysis in the presence of NaCl–DOX@PLGA nanoparticles. No obvious hemolysis was observed in any of the experimental or negative control groups, whereas the positive control group showed significant hemolysis. On the basis of these results, we conclude that NaCl–DOX@PLGA nanoparticles have low toxicity and good biocompatibility.

### Effects of DOX and MW on intracellular Ca^2+^ concentration

**Fig. 3:**
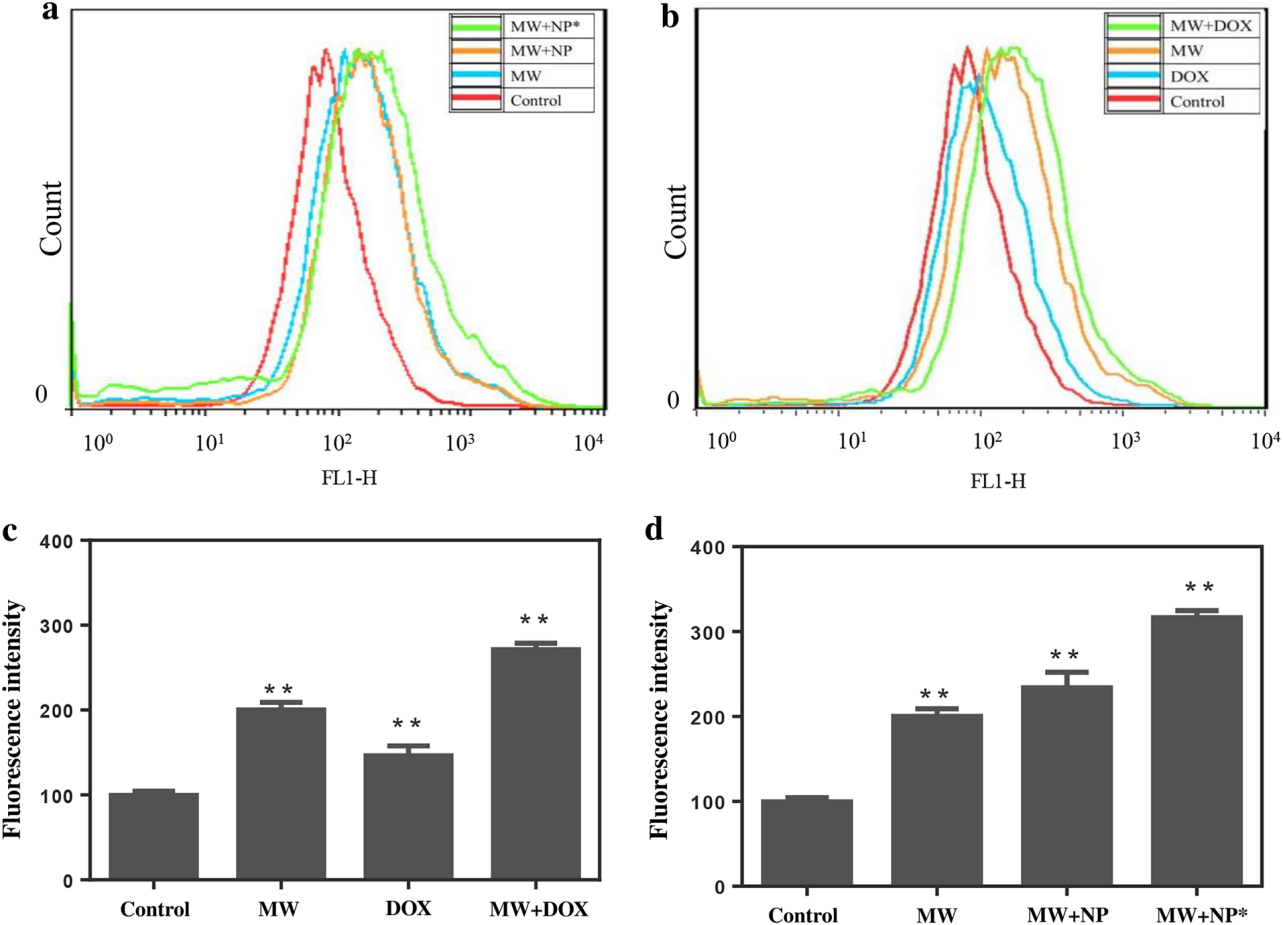
The intracellular Ca^2+^ concentration under different treatment conditions. **a** Intracellular Ca^2+^ concentration in control(red), MW (blue), MW + NP (orange) and MW + NP* (green). **b** Intracellular Ca^2+^ concentration in control(red), DOX (blue), MW (orange) and MW + DOX(green). The concentration of Ca^2+^ in DOX + MW group was highest. **c** The four histograms represented the mean fluorescence intensity in the control, DOX, MW and DOX + MW group. **d** The four histograms represented the mean fluorescence intensity in the control, MW, MW + NP and MW + NP* group. Significant differences were found in all experimental group when compared with the control group.* NP* NaCl@PLGA nanoparticles, NP* NaCl–DOX@PLGA nanoparticles, **P < 0.01

DOX is a common antitumor drug that is widely used in cancer chemotherapy. Its biological effects are due to binding to DNA-associated enzymes and intercalating the base pairs of the DNA double helix [31]. It can also induce free radical generation, which can cause cellular damage [32]. Several studies have proved the interplay between Ca^2+^ and free radical generation [33]. Large surges in intracellular Ca^2+^ could initiate apoptosis and elicit cell death [34–36]. We have validated that a small dose of DOX could increase the Ca^2+^ concentration in HepG2 cells, and mild MW irradiation has a similar effect. Furthermore, both effects did not cause obvious damage to cell viability. When the HepG2 cells were incubated with DOX for 12 h and subsequently irradiated with mild MW, the mean fluorescence intensity of intracellular Ca^2+^ showed a significant increase compared with cells that were only treated once, as the flow cytometry results showed a shift of the curve to the right (Fig. 3a, c). To further evaluate the effect of an increase in intracellular Ca^2+^ on apoptosis of HepG2 cells, the cells were stained with Annexin V-FITC/PI, and the effects were detected using flow cytometry. Results revealed that the increase in Ca^2+^ concentration dramatically triggered an apoptotic response in HepG2 cells compared with the only MW and DOX group. The percentage of apoptotic/necrotic cells in the DOX and MW groups was 10.12% and 28.63%, respectively. However, the percentage of apoptotic/necrotic cells increased to 40.7% after the combined treatment of DOX and MW irradiation (Fig. 5a).

Inspired by these results, we further loaded DOX into the PLGA nanoparticles to create the NaCl–DOX@PLGA nanoplatform. The creation of the nanoplatform provided an opportunity to eliminate the undesirable side effects and poor targeting of DOX and to greatly improve the therapeutic effect of this chemotherapeutic treatment. We assessed the effect of NaCl–DOX@PLGA nanoparticles on the concentration of intracellular Ca^2+^ after the release of DOX from the nanoparticles was confirmed. The HepG2 cells were incubated with NaCl–DOX@PLGA nanoparticles for 12 h to guarantee sufficient uptake. Afterwards, the cells were gently washed with PBS to remove the extracellular nanomaterials and were subsequently irradiated with mild MW (2 W for 2 min). Flow cytometry results showed that the concentration of intracellular Ca^2+^ treated with both nanoparticles and MW increased significantly compared with that in cells irradiated with the MW or nanoparticles alone (Fig. 3b, d). The mean fluorescence intensity was 99.7 ± 4.7, 123 ± 13.6, and 316 ± 8.2 in the control, nanoparticle, and combination groups, respectively. These results indicate that mild MW irradiation can increase the Ca^2+^ concentration in HepG2 cells pretreated with NaCl–DOX@PLGA nanoparticles.

### MMP changes after pretreatment with nanoparticles and MW irradiation

**Fig. 4:**
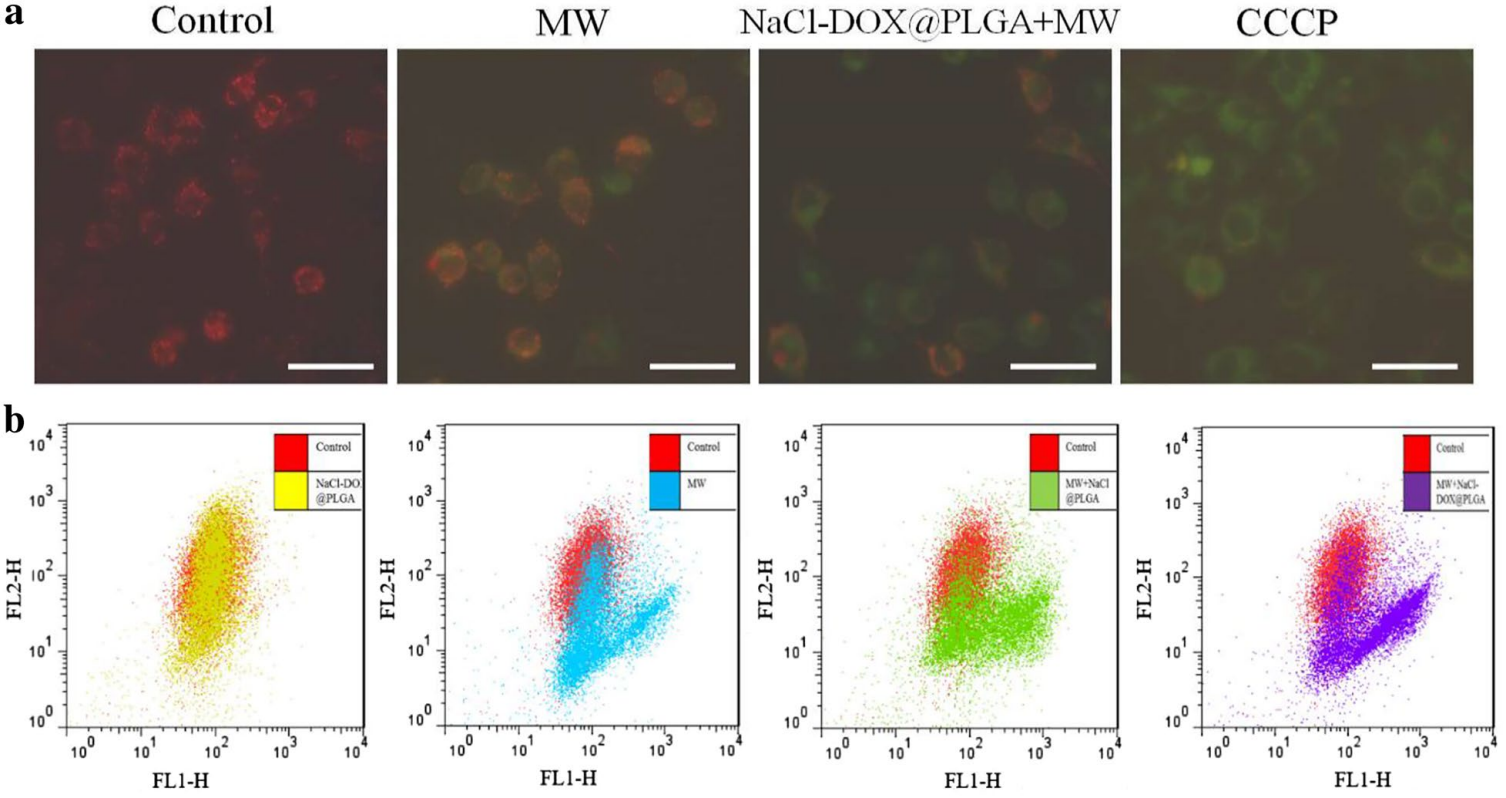
Fluorescence changes of HepG-2 cells in different groups. **a** Fluorescence microscopy images showed the MMP dissipation of HepG-2 cells after the pretreatment of nanoparticles and MW irradiation. **b** Flow cytometry results of MMP changes pretreated with DOX, NaCl–DOX@PLGA nanoparticles or/and irradiated with MW. Red: control; yellow: NaCl–DOX@PLGA; blue: MW irradiation; green: MW + NaCl@PLGA; purple: MW + NaCl@PLGA. The scale bar is 50 μm

MMP changes after the pretreatment of cells with nanoparticles and MW irradiation were investigated using JC-1 staining. JC-1, which is released from mitochondria into the cytoplasm, will accumulate along the mitochondrial membrane and emits an orange–red fluorescence signal lower than the 488 nm excitation wavelength when the MMP increases. MMP is known to decrease during apoptosis, and in this case, the JC-1 monomer will emit a green signal. Pretreatment of the HepG2 cells with NaCl–DOX@PLGA nanoparticles for 12 h caused no obvious change in MMP, which was shown as an emission of red fluorescence (Fig. 4a). MW irradiation alone of the HepG2 cells caused a decrease in MMP, which was shown as an increase in green fluorescence. After the combined treatment of NaCl–DOX@PLGA nanoparticles and MW irradiation, more obvious dissipation of MMP was detected as a greater increase in green fluorescence in the JC-1 staining image (Fig. 4a). Subsequently, the flow cytometer results provided the quantitative changes of MMP after different treatments. In response to treatment with MW irradiation, the percentage of JC-1-emitted green cells increased by 23.1% compared with that in the control group, whereas MMP changes were found after culture of NaCl–DOX@PLGA nanoparticles, which were shown as overlap cell population in the flow cytometer results (Fig. 4b). Cells pretreated with the combination of NaCl–DOX@PLGA nanoparticles and MW irradiation resulted in a greater percentage of MMP dissipation than in cells with the combined treatment of DOX and MW irradiation (52.6% vs. 37.4%), which was illustrated as more cells shifting to the upper right and lower right quadrants of the fluorescence-activated cell-sorting display (Fig. 4b). These results showed that the combination of NaCl–DOX@PLGA nanoparticles and MW irradiation caused a significant decrease in MMP in the HepG2 cells, which may ultimately lead to an increase in apoptosis.

### Cellular viability evaluation after combined treatment

**Fig. 5:**
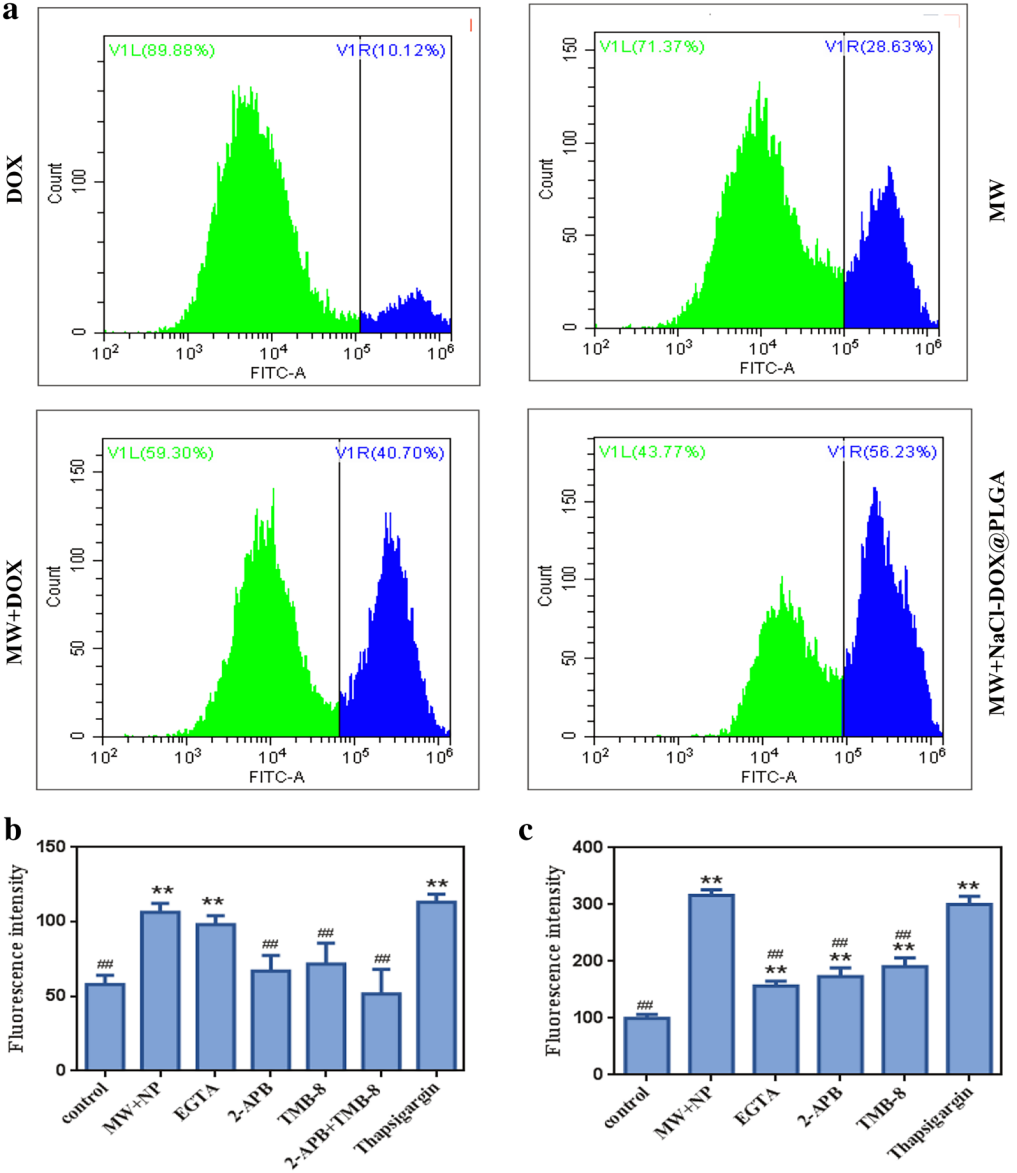
**a** Flow cytometry results of HepG-2 cells stained by Annexin V-FITC/PI. **b** Ca^2+^ fluorescence intensity induced by MW after the pre-treatment of EGTA, 2-APB, TMB-8, and thapsigargin. **c** Ca^2+^ fluorescence intensity induced by MW combined with nanoparticles after the pre-treatment of EGTA, 2-APB, TMB-8, and thapsigargin

We further evaluated cellular viability after the combined treatment of cells with NaCl–DOX@PLGA nanoparticles, and MW irradiation was shown to induce an increase in intracellular Ca^2+^ and decrease in MMP. Apoptosis or cell death occurred in approximately 10% of cells after incubation of NaCl–DOX@PLGA nanoparticles. The percentage of apoptotic or dead cells was approximately 28% and 40% in the MW and MW + DOX groups, respectively, whereas the percentage reached 56% when the cells were treated with NaCl–DOX@PLGA + MW (Fig. 5a). The combined treatment of mild MW irradiation and NaCl–DOX@PLGA nanoparticles was shown to lead to more apoptosis or cell death for tumor cells.

The relationship between cell apoptosis/death and Ca^2+^ concentration was initially analyzed. Evidence gathered from the past 10 years showed the importance of Ca^2+^ in the activation and execution of cell death [37, 38]. Intracellular Ca^2+^ increases have been shown to be required for apoptosis to occur [39]. The mitochondria is central to cell death [38]. The increases in intracellular Ca^2+^ concentration cause the activation of mitochondrial membrane permeabilization, interruption of respiratory chain functions, and loss of MMP, which will result in apoptosis and necrosis [40]. Our results confirmed that the increase in intracellular Ca^2+^ concentration caused by combined treatment can lead to dissipation of MMP. More apoptosis and dead tumor cells were detected in groups with higher intracellular Ca^2+^ concentration and lower MMP. Tumor cells become more sensitive to MW treatment after pretreatment with DOX, and this may be attributed to the nonthermal effects of MW exposure. However, this possibility needs more evidence.

Recently, the role of the non-thermal effects of MW in the synthetic treatment of cancer has gained attention. Long et al.’s study have suggested that the nonthermal effects of MW irradiation can induce cellular uptake of therapeutic agents [9]. Yu et al. have found that nonthermal effects of low-power microwave radiation can cause apoptosis in the epithelial cells of the lens [41], but no studies have illustrated the role of nonthermal MW on the intracellular Ca^2+^ concentration in tumor cells. MW irradiation may cause polarization and electronic vibrations of cellular membranes. This process might affect the function of the ER–mitochondrial interface and plasma membrane, which lies at the center of Ca^2+^ homeostasis [42]. Coordinated reactive oxygen/nitrogen species (ROS/RNS) and Ca^2+^ surges are required for the initiation of apoptosis at the ER–mitochondrial interface [33]. ROS is an important factor produced during photodynamic therapy and could induce cell death through apoptosis and/or necrosis pathways, tumor vasculature damages, Brandes et al. [43], Lismont et al. 44]. Mild MW irradiation may also have some influence on ROS production in tumor cells, but this needs further study.

### Effect of EGTA, 2-APB, TMB-8, and thapsigargin on MW with nanoparticle-induced Ca^2+^ concentration in HepG2 cells

Increased intracellular Ca^2+^ concentration in non-excitable cells mainly originates from two pathways. These are Ca^2+^ release from the ER Ca^2+^ stores and plasmalemmal Ca^2+^ entry via store-operated Ca^2+^ entry. We investigated the possible mechanisms underlying the combined treatment-induced Ca^2+^ changes and related apoptosis by using the extracellular Ca^2+^ chelator EGTA, store-operated Ca^2+^ channel (SOC) inhibitor 2-APB, ER Ca^2+^-release-antagonist TMB-8, and sarco/ER-Ca^2+^-ATPase inhibitor thapsigargin. EGTA, 2-APB, TMB-8, and thapsigargin were cultured with cells before MW irradiation. TMB-8 significantly inhibited the increase in intracellular Ca^2+^ induced by MW irradiation (P < 0.05), whereas no significant inhibitions were found in cells cultured with EGTA, 2-APB, and thapsigargin (P > 0.05) (Fig. 5b). Different results were found in cells cultured with the abovementioned inhibitors and nanoparticles before MW irradiation. EGTA, 2-APB, and TMB-8 significantly decreased the concentration of intracellular Ca^2+^ after cells were cultured with nanoparticles and exposed to MW irradiation. No change was observed for thapsigargin (Fig. 5c). Our results showed that the increase in intracellular Ca^2+^ concentration caused by MW irradiation may be achieved by Ca^2+^ release from the ER. The increase in Ca^2+^ concentration induced by the combination of nanoparticles and MW irradiation was shown to be a more complicated pathway, including the release of Ca^2+^ from both external and internal sources.

#### Safety evaluation in vivo

**Fig. 6:**
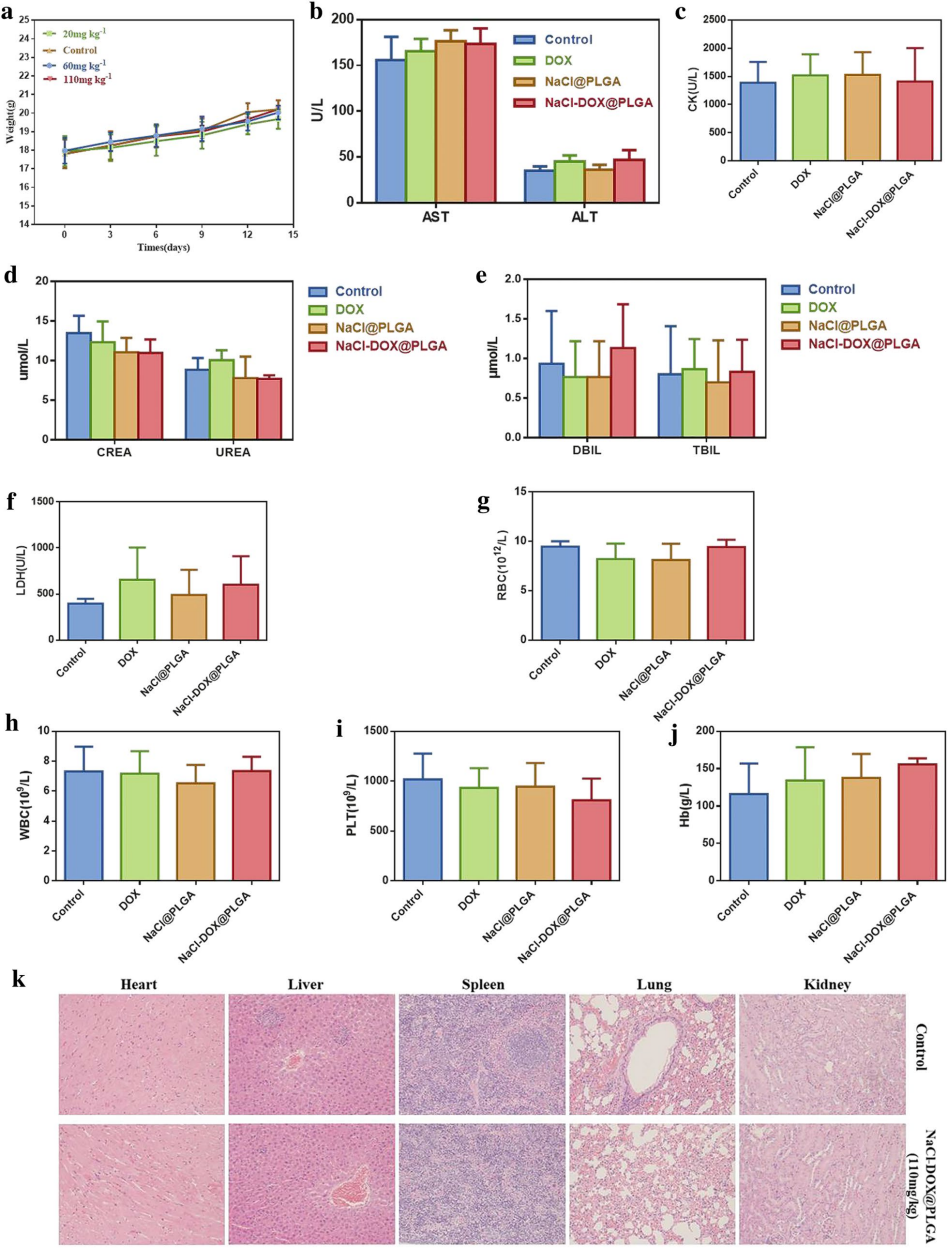
Safety evaluation in vivo of the NaCl–DOX@PLGA nanoparticles at different concentrations (20, 60, 110 mg/kg; PBS-treated nude mice were set as the control). **a** Body weight change curve for 14 days. **b**–**f**. Blood routine including ALT, AST, CK, DBIL, TBIL, UREA, and CREA; **g**–**j** blood biochemistry including RBC, WBC, PLT, and Hb of each group. **k** HE staining assay of liver, spleen, lung, kidney and heart were harvested form different groups (110 mg/kg and PBS control group). The scale bar is 20 μm

No significant difference was found in the body weight of nude mice between groups exposed to different concentrations of NaCl–DOX@PLGA nanoparticles (20, 60, and 110 mg/kg) or the PBS control, and the body weight showed a slow upward trend in each group (Fig. 6a). Biochemistry and blood routine experiments were performed to further verify the toxicity of the NaCl–DOX@PLGA nanoparticles to nude mice. The biochemical parameters investigated were red blood cells (RBC), white blood cells(QBC), platelet(PLT), hemoglobin(Hb), alanine aminotransferase(ALT), aspartate aminotransferase(AST), creatine kinase(CK), direct bilirubin(DBIL), total bilirubin(TBIL), urea(UREA), and creatinine(CREA). Compared with the control group, nude mice in all experimental groups did not show any obvious abnormalities in any of these parameters (Fig. 6b–j). The H&E-stained images (Fig. 6k) of the main organs (liver, spleen, lung, kidney, and heart) collected from the DOX, NaCl@PLGA, and NaCl–DOX@PLGA groups indicated no obvious tissue denaturation or organ damage in the administered mice. No significant change was found in heart slices of the DOX and NaCl–DOX@PLGA groups despite the most common side effect of DOX being cardiac toxicity. These results confirmed that the materials tested were not toxic to the mice even at a high dose of 100 mg kg^−1^.

### In vivo antitumor efficacy evaluation of NaCl–DOX@PLGA combined with MW ablation

**Fig. 7:**
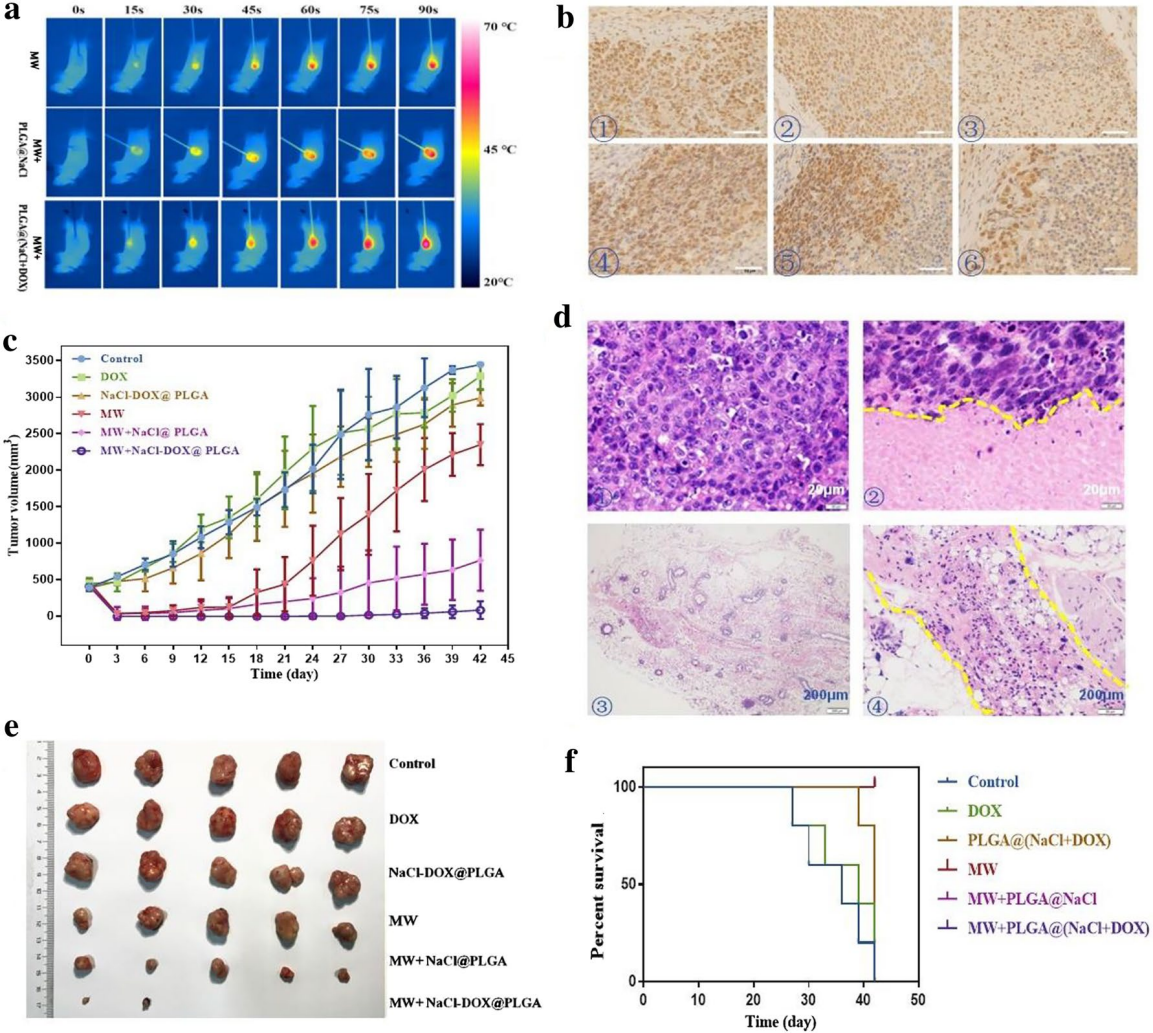
In vivo outcomes of MW irradiation. **a** FLIR images monitoring temperature changes of tumors site during the MW ablation. **b** PCNA staining results in the peripheral ablation area. ①: control, ②: free DOX, ③: NaCl–DOX@PLGA, ④: MW, ⑤: MW + NaCl@PLGA, ⑥: MW + NaCl–DOX@PLGA. The scale bar is 50 μm. **c** tumor volume growth curve in different groups.** d** HE results of control tumor (①), local tumor progression (②), and complete ablation (③–④).** e** The tumor harvested at the end of observation.** f** survival rate curve of nude mice

Thermal ablation has been recommended as an important curative therapy for early-stage liver cancer. Efficient thermal energy is critical to achieve complete ablation. Excess energy might cause damage to the surrounding normal tissues and lead to severe complications. Maintaining therapeutic efficacy while reducing the output energy is critical in precise ablation. A solution to achieve this purpose would be to enhance the acceptability of tumor cells to ablation at lower output energy conditions. We have illustrated that nonthermal effects of MW irradiation could significantly increase the intracellular Ca^2+^ concentration caused by DOX in ex vivo studies. More tumor cells were killed because of the combined effect of DOX and the nonthermal effect. This combination was deemed to be safe because the DOX concentration had low toxicity, and the output energy of MW was mild. This combination effect was discovered in the in vivo experiments described in this study. It was shown that MW assisted by NaCl@PLGA nanoparticles could significantly enlarge the ablation volume but did not completely kill the tumor cells in spatial dimension (Additional file 1: Figure S5). This was in agreement with in vitro experiments, which found that MW irradiation combined with DOX had a synergistic effect on the surge of cytosolic Ca^2+^, thus enhancing the antitumor effect of chemotherapy and simultaneously enhancing the sensitivity of the tumor to MW irradiation. PLGA plays an important role in the delivery of drugs in a controlled and targeted manner [30]. In our study, the PLGA nanoparticles showed good potential as a drug carrier, and they showed some MW-stimuli-controlled response and accelerated DOX release under MW stimulation. Thus, we finally achieved a biocompatible MW-stimuli-controlled-release of NaCl–DOX@PLGA nanoparticles that integrated thermal effects, nonthermal effects, and chemotherapy. A HepG2 hepatic cancer tumor model was established in BALB/c nude mice to evaluate the inhibition effect of the NaCl–DOX@PLGA nanoparticles on tumor growth. Healthy female mice were randomly assigned into six groups and injected with free DOX, NaCl@PLGA, and NaCl–DOX@PLGA via the tail vein. The mice in the MW, MW + NaCl@PLGA, and NaCl–DOX@PLGA + MW groups were treated with MW ablation at the tumor site at a low power of 2 W, 2450 MHz for 1.5 min 6 h post-injection, whereas the other nude mice underwent no further treatment. The output energy in this study was lower than the energy required to completely ablate the tumor in one session. In clinical practice, excess energy in one ablation session might cause severe complications [45]. Hence, exploring methods to reduce output power and shorten ablation time is necessary. Because of the multifunctional platform, the tumor cells were more sensitive to MW ablation and chemotherapy. The loaded nanoparticles provided an efficient and safe solution enhancing MW efficacy and therapy outcomes. The real-time infrared thermal images (Fig. 7a) of the tumor site visually displayed the process of incremental temperature increases in different groups. The increase in temperature at the tumor site by MW irradiation was time dependent, and the rate of temperature increase was higher in the MW + NaCl@PLGA and the NaCl–DOX@PLGA + MW group than that in the MW group. NaCl is a common clinical reagent that shows a high thermal conversion efficacy under MW irradiation when incorporated in nanoparticles. During the irradiation process, the temperature of the tumor site in the MW + NaCl–DOX@PLGA group increased to 62 °C in the MW + NaCl@PLGA group, whereas the control group only reached 56.3 °C. The temperature differences between the MW + NaCl–DOX@PLGA and MW + NaCl@PLGA groups were not significant. The thermal effect of MW increased the temperature, the NaCl solution accelerated the ablation process, and the PLGA rapidly released DOX at the tumor site under MW stimuli. The cellular uptake of DOX simultaneously had a synergistic effect with MW irradiation in inducing cell apoptosis or cell death. Long et al.’s study has illustrated that the nonthermal effect of MW irradiation could promote the cellular uptake of nanoparticles and anticancer drugs. The increased uptake of DOX in this study may enhance not only chemotherapy but also MW irradiation. In the ablation process, particularly using the needle-like antenna, the center of the ablation area achieved complete coagulation because thermal energy was emitted from the tip of the antenna and was disseminated gradually to the distant tumor tissues. Therefore, the temperature of the peripheral area was the lowest, which could result in local tumor progression [6]. The loaded nanoparticles provided a feasible solution to overcome this problem. The NaCl–DOX@PLGA nanoparticles were shown to enhance the sensitivity of tumor cells to MW in a nonlethal thermal environment. Consequently, more tumor cells would be killed at temperatures even less than 45 °C. The animal studies validated the synergistic effect of MW ablation and NaCl–DOX@PLGA nanoparticles. Three mice in each group were killed 3 days after ablation to obtain tumor tissues for analysis of the therapeutic effect at an early stage. Histological analysis revealed that tumor cells in the center of the ablation area showed coagulative necrosis. Cell apoptosis and proliferation were then assessed in the peripheral ablation area by TUNEL (Additional file 1: Figure S6a) and PCNA staining (Fig. 7b). Treatment with free DOX or NaCl–DOX@PLGA nanoparticles alone induced < 20% apoptotic cells, both with no significant differences compared with the control group. The MW, MW + NaCl@PLGA, and MW + NaCl–DOX@PLGA groups showed significant differences in the apoptosis index (AI) (P < 0.05) compared with the control group. The combination of MW and NaCl–DOX@PLGA nanoparticles induced the highest level of AI, and > 85% tumor cells developed apoptosis, which was significantly higher than that induced by treatments of MW + NaCl@PLGA or MW alone (*P* < 0.05). We also found that the PCNA staining intensities had significantly decreased in the MW, MW + NaCl@PLGA, and MW + NaCl–DOX@PLGA groups, but the most significant decrease was found in the MW + NaCl–DOX@PLGA group (P < 0.05). Only a small fraction of PCNA-positive cells was detected in the periphery of the ablation area.

Additional file 1: Figure S7 shows that the body weight of nude mice increased during the experimental period, whereas the body weight in the MW, MW + NaCl@PLGA, and MW + NaCl–DOX@PLGA groups decreased 3 days after ablation and slowly increased afterwards. It was attributed to a compensative response of the body after tumor ablation. On days 18–21, the body weight had a tendency to decrease again. Local tumor progression was most frequently detected during this period. The increasing tumor burden may be the reason for the second decline in body weight. The DOX-loaded group (NaCl–DOX@PLGA with and without MW irradiation groups) did not indicate an obvious loss in body weight, which further validated the stability of DOX in the NaCl–DOX@PLGA platform and the good compatibility of the loaded nanoparticles.

After 42 days of observation, the NaCl–DOX@PLGA group expressed a significant suppression in tumor growth than the other two groups among all nude mice treated with MW irradiation. Figure 7c shows that the mean tumor volume was 2351 and 760 mm^3^ in the MW and NaCl@PLGA + MW groups, respectively, whereas it reduced to 84 mm^3^ in the NaCl–DOX@PLGA + MW group. The mean tumor volume in nude mice without MW irradiation was approximately 3000 mm^3^, which indicated that NaCl–DOX@PLGA alone did not cause an obvious inhibitive effect on tumor growth. The tumor volume reached 760 mm^3^ in the NaCl@PLGA group versus 2351 mm^3^ in the MW alone group, which illustrated that NaCl@PLGA may help produce more energy to damage tumor cells under mild MW, but the effect was very limited. After loading DOX to the NaCl@PLGA nanoparticles, the DOX was not only used as a chemotherapeutic agent but also acted as a medium to enhance the nonthermal effect of MW ablation. DOX could sensitize tumor cells to MW irradiation and further induce more occurrences of tumor cell death, particularly for cells in the periphery of the ablation area. The representative photos of mice shown in Additional file 1: Figure S8 and Fig. 7d taken at different times, before and after, treatment illustrated the typical tumor development and were consistent with the results of tumor volume growth curves. A residual tumor was found in 60% of mice immediately after treatment in the MW group, and tumor progression was found in the rest of the mice on the 18th day after ablation. Obvious residual tumor in 80% of mice in the NaCl@PLGA + MW group was not found in the early period, whereas tumor progression was found between the 9th and 33rd days. In the NaCl–DOX@PLGA + MW group, all mice showed complete ablation in the early period of observation. Tumor progression was found in one mouse on the 24th day and one mouse on the 30th day. Obvious tumor progression was found later, but the speed of tumor growth in the NaCl–DOX@PLGA + MW group was slower than that in the two groups that received MW treatment.

At the end of observation, all nude mice died in the control and free DOX groups. In the NaCl–DOX@PLGA group, 40% of the mice were still alive, but the maximum tumor size was approximately 20 mm. Tumors harvested at the end of the experiment are shown in Fig. 6e. All nude mice were alive in groups that received MW treatment, and the final survival rate was 100% (Fig. 7f). H&E-stained images of the heart, liver, spleen, kidney, and lung harvested at the end (Additional file 1: Figure S9) illustrated good compatibility of the as-made nanoparticles. The outcomes demonstrated that combined chemotherapy and MW (nonthermal and thermal) therapies based on a NaCl–DOX@PLGA nanoplatform could significantly improve the survival rate of HepG2-tumor-bearing mice.

## Conclusions

A biocompatible and biodegradable NaCl–DOX@PLGA nanoplatform was prepared with triple functions to realize the effective tumor killing under unlethal temperature. The as-made nanoplatform developed in this study was shown to significantly enlarge the ablation volume. The elevated intracellular Ca^2+^ caused by MW irradiation and DOX destroyed the homeostasis of tumor cells, inducing a decrease in MMP and massive apoptosis. This process can make tumor cells more sensitive to MW ablation and DOX chemotherapy, which could provide many clinical benefits by decreasing the output energy of MW and reducing the concentration of DOX used. The development of nano-scale microwave-sensitive agents to kill tumors in unlethal temperature reduced the dependence of ablation on high temperature and greatly improve the safety of ablation. The versatility of this unique nanoplatform provides promising biosafe therapeutic nanoagents for the non-invasive treatment of risk-location tumors in vivo.

## Supplementary information

**Additional file 1: Figure S1.** Ultrasound-guided percutaneous insertion of the MW antenna into the tumor center in real-time.** Figure S2.** The hydrodynamic diameter and zeta-potential of NaCl@PLGA nanoparticles.** Figure S3.** Temperature changes in different groups.** Figure S4.** Cellular uptake of PLGA nanoparticles.** Figure S5.** TTC staining results of tumor tissues after MW ablation.** Figure S6.** TUNEL staining results in the peripheral ablation area.** Figure S7.** Body weight change curve in different groups.** Figure S8.** Three different kinds of tumor development observed in experimental period.** Figure S9.** HE staining of liver, spleen, lung, kidney, and heart of nude mice at the end of observation.

## Data Availability

All data generated or analyzed during this study are included in this published article and its additional file.

## Abbreviations

MW: microwave
DOX: doxorubicin hydrochloride
PLGA: poly(lactic-co-glycolic acid)
NaCl: sodium chloride
DMSO: dimethyl sulfoxide
PVP: polyvinylpyrrolidone
SEM: scanning electron microscopy
EDS: energy-dispersive spectroscopy
FLIR: forward-looking infrared
PBS: phosphate-buffered saline
HBSS: Hank's Balanced Salt Solution
JC-1: 5,50,6,60-tetrachloro-1,10,3,30-tetraethylbenzimidazolylcarbocyanine iodide
FACS: fluorescence-activated cell-sorting
DAPI: 4′,6-diamidino-2-phenylindole
ER: endoplasmic reticulum
2-APB: 2-aminoethoxydiphenyl borate
TMB-8: 3,4,5-trimethoxybenzoic acid 8-(diethylamino) octyl ester
SERCA: sarco/ER-Ca^2^^+^-ATPase inhibitor
ROS/RNS: reactive oxygen/nitrogen species
RBC: red blood cells
QBC: white blood cells
PLT: platelet
Hb: hemoglobin
ALT: alanine aminotransferase
AST: aspartate aminotransferase
CK: creatine kinase
DBIL: direct bilirubin
TBIL: total bilirubin
CREA: creatinine
H&E: hematoxylin and eosin
