# Supplementary material for: Amplified intracellular Ca2+ for synergistic anti-tumor therapy of microwave ablation and chemotherapy
Source: J Nanobiotechnology. 2019 Dec 2;17:118. doi: 10.1186/s12951-019-0549-0 (PMC6889637; doi:10.1186/s12951-019-0549-0)
Supplement: Supplementary file 1 — Additional file 1: Figure S1. Ultrasound-guided percutaneous insertion of the MW antenna into the tumor center in real-time. Figure S2. The hydrodynamic diameter and zeta-potential of NaCl@PLGA nanoparticles. Figure S3. Temperature changes in different groups. Figure S4. Cellular uptake of PLGA nanoparticles. Figure S5. TTC staining results of tumor tissues after MW ablation. Figure S6. TUNEL staining results in the peripheral ablation area. Figure S7. Body weight change curve in different groups. Figure S8. Three different kinds of tumor development observed in experimental period. Figure S9. HE staining of liver, spleen, lung, kidney, and heart of nude mice at the end of observation. [file 12951_2019_549_MOESM1_ESM.docx]

Additional Information

**Synergistic Nanoplatform for Amplifying Intracellular Calcium to Sensitize Tumor Cells to Microwave Ablation and Chemotherapy**

*Jian-ping Dou^1^, Qiong Wu^2^, Chang-hui Fu^2^, Dong-yun Zhang^1^, Jie Yu^1^*, Xian-wei Meng^2^*, Ping Liang^1^**

**Additional Figures**


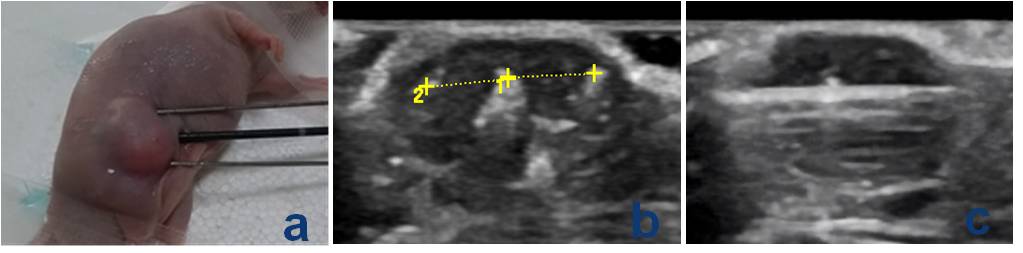


Figure S1. Ultrasound-guided percutaneous insertion of the MW antenna into the tumor center in real-time. (a) MW antenna was placed in the center and two thermal needles were placed 5mm apart from the antenna. The transverse (b) and the vertical section (c) of antenna and thermal needle.

a
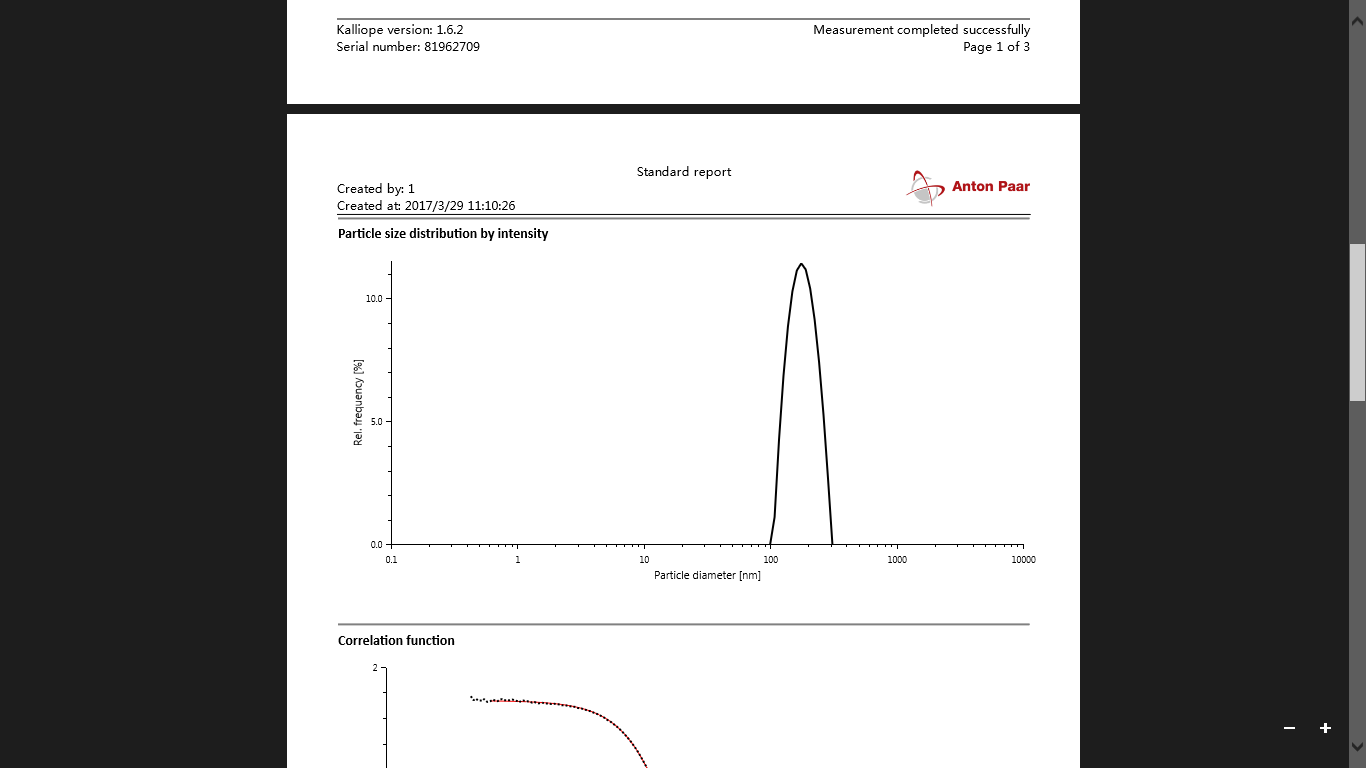


b
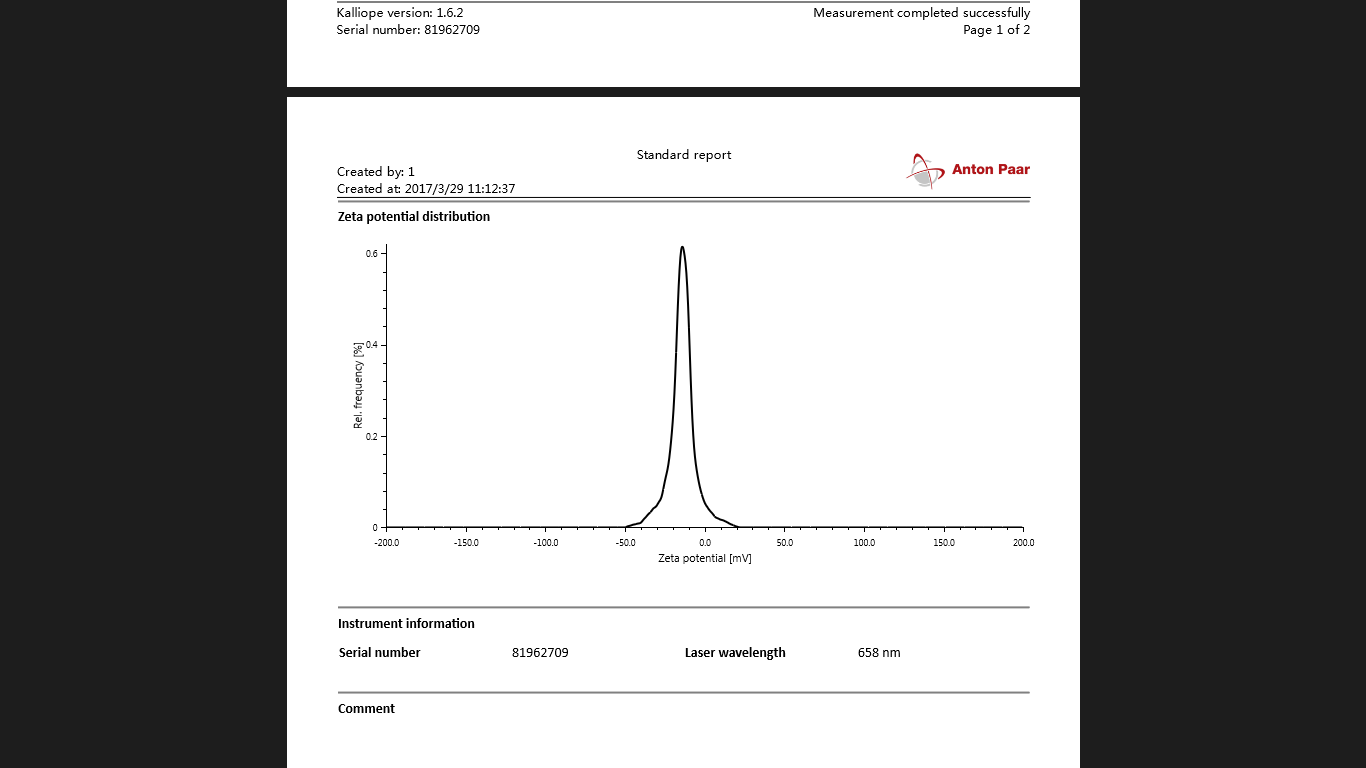


Figure S2. The hydrodynamic diameter and zeta-potential of NaCl@PLGA nanoparticles(a-b).

Figure S3. Temperature changes in different groups.


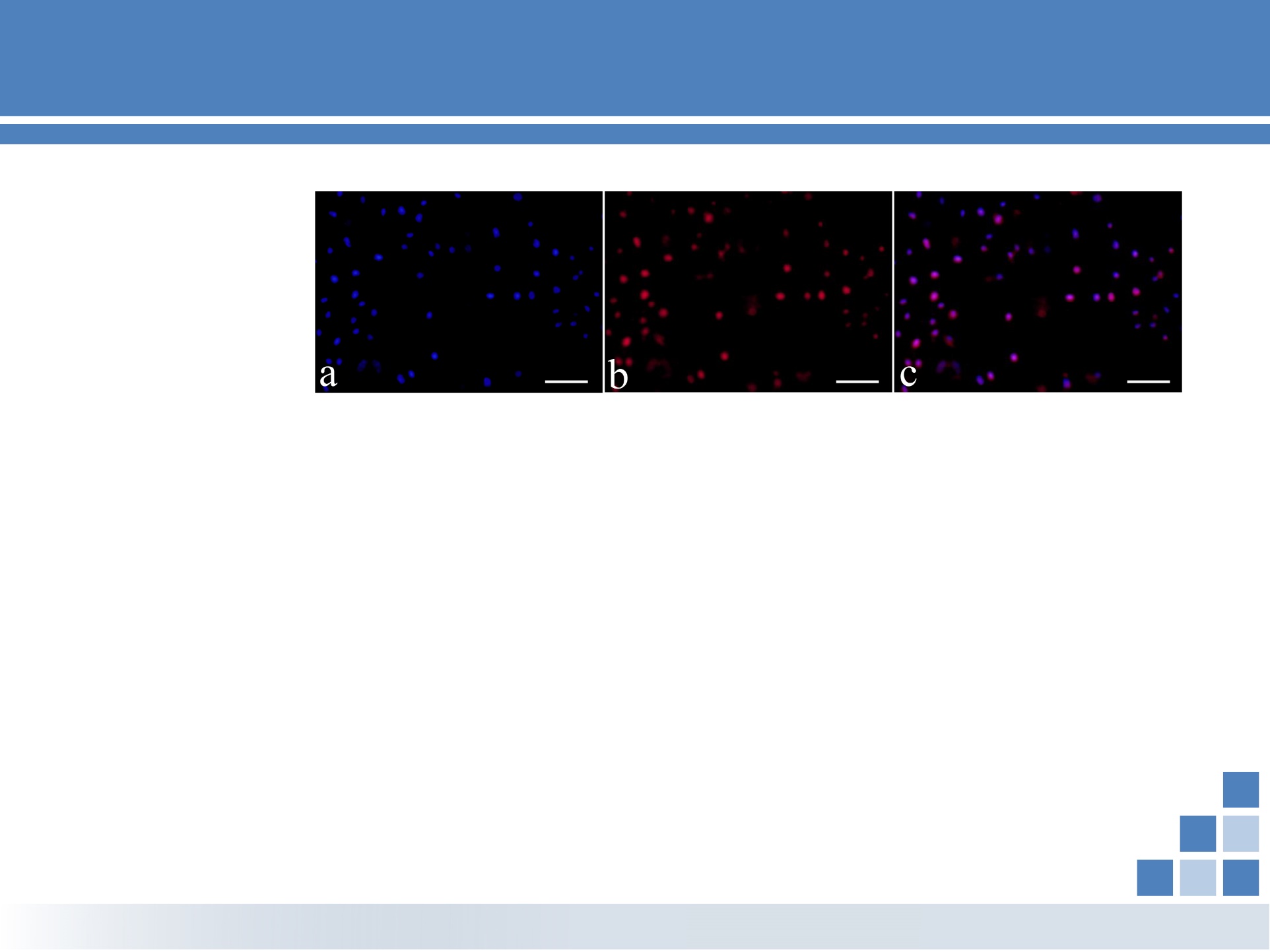


Figure S4. Cellular uptake of PLGA nanoparticles. a.DAPI; b. rhodamine 6G; c.merge. The scale bar is 50μm.


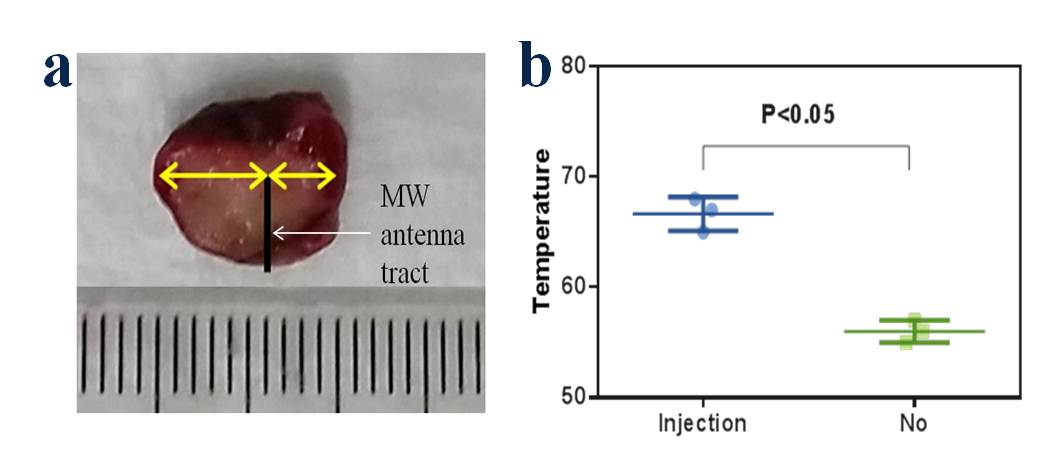

Figure S5. TTC staining results of tumor tissues after MW ablation. a. the MW antenna was insert into the center of the tumor. NaCl@PLGA nanoparticles were injected into the right peripheral tumor 5mm apart from the center, while the left was set as a control and no solution was injected. B. The mean maxium diameter was 56±2.2mm in the right side and 35±2.0mm in the left side.


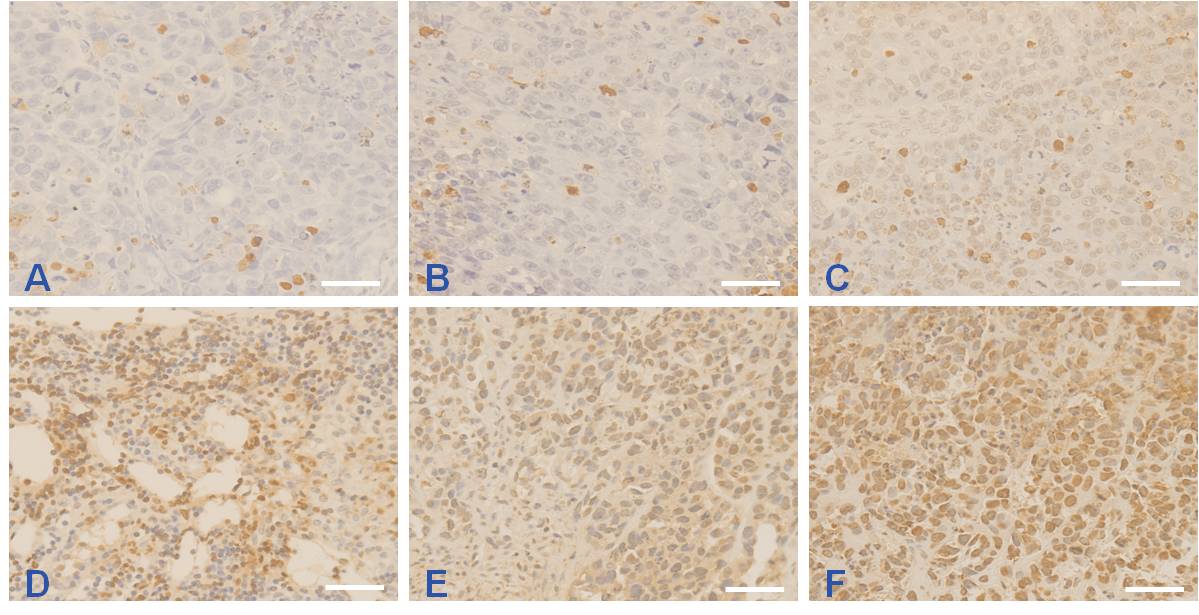


Figure S6. TUNEL staining results in the peripheral ablation area. A：control, B：free DOX, C：NaCl-DOX@PLGA, D：MW, E：MW+NaCl@PLGA, F：MW+NaCl-DOX@PLGA. The scale bar is 50μm


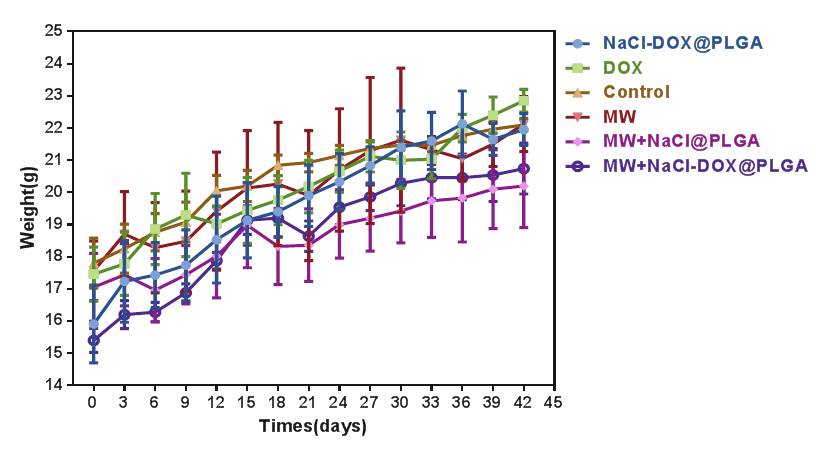


Figure S7. Body weight change curve in different groups.


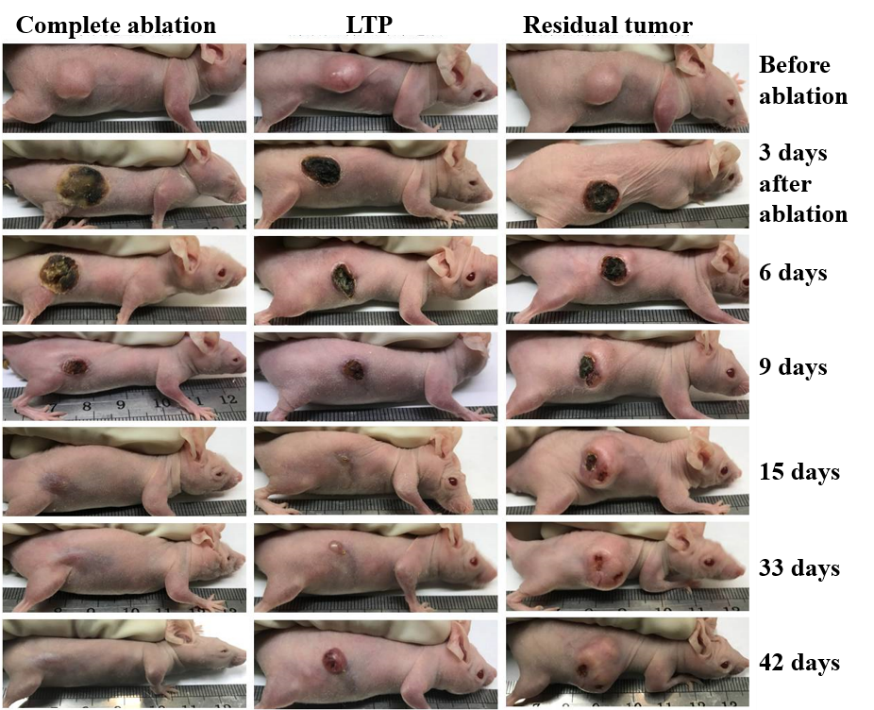


Figure S8. Three different kinds of tumor development observed in experimental period. LTP: local tumor progression.


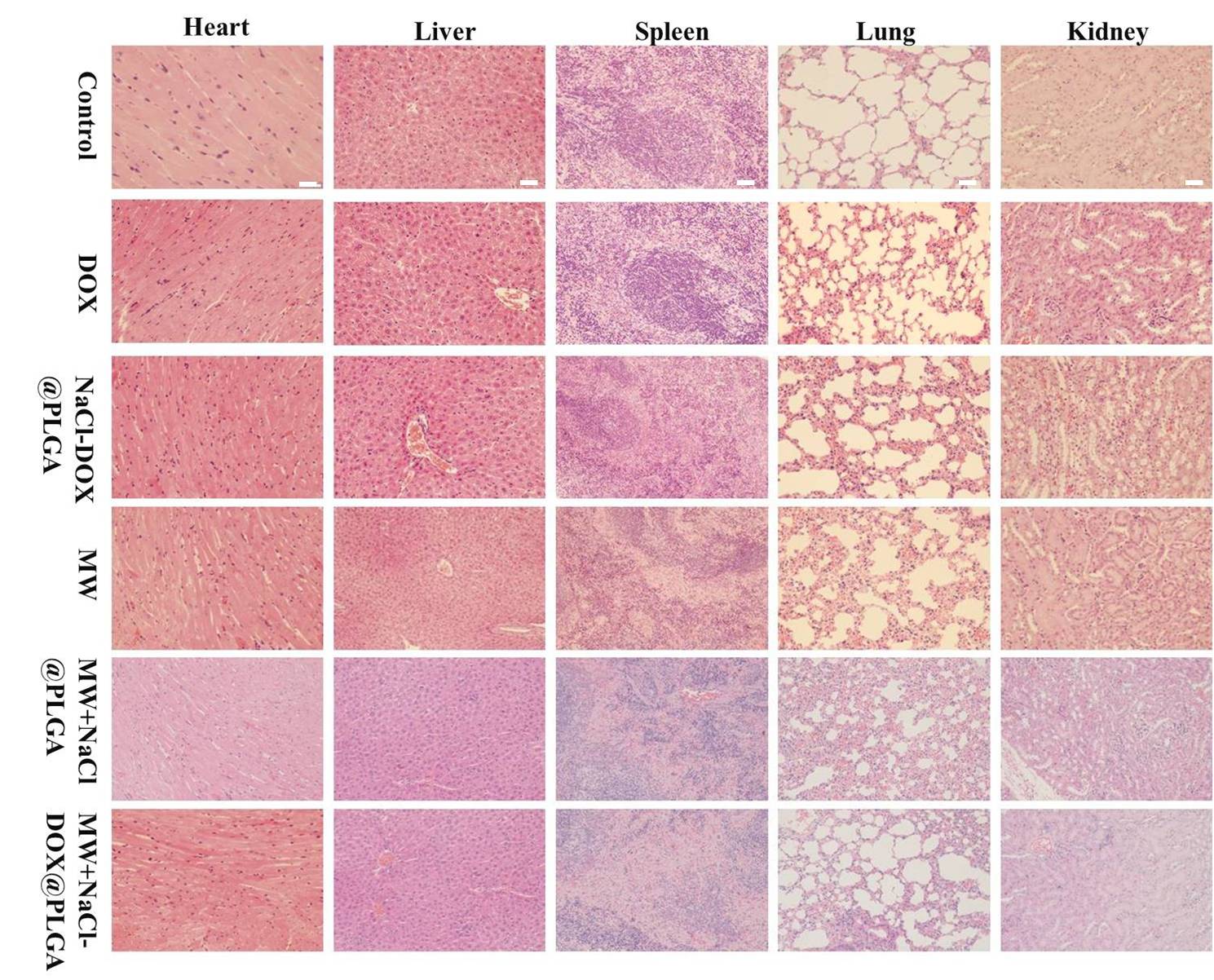


Figure S9. HE staining of liver, spleen, lung, kidney, and heart of nude mice at the end of observation. All scale bars are 20μm.
